# Peripartum sertraline impacts maternal neurobehavioral and neurodegenerative mechanisms in pregnant and postpartum mice

**DOI:** 10.1038/s41380-025-03094-x

**Published:** 2025-07-18

**Authors:** Brianna Blaine, Mushroor Kamal, Mizani Roberts, Brandon Schickling, Marisol Lauffer, Yuping Zhang, Aimee Bertolli, Matthew A. Weber, Robert Taylor, Sana Nadeem, Krushi Patel, Lynn Teesch, Georgina Aldridge, Donna Santillan, Mark Santillan, Serena Gumusoglu

**Affiliations:** 1 Iowa Neuroscience Institute, Iowa City, IA USA; 2 University of Iowa Department of Obstetrics and Gynecology, Iowa City, IA USA; 3 University of Iowa Department of Neurology, Iowa City, IA USA; 4 University of Iowa Department of Psychiatry, Iowa City, IA USA; 5 University of Iowa Department of Chemistry, Iowa City, IA USA

**Keywords:** Neuroscience, Molecular biology

## Abstract

Selective Serotonin Reuptake Inhibitors (SSRIs) are among the most common medications used for depression in postpartum and lactating people, who experience increased depression risk. However, there is a limited understanding of peripartum SSRI impacts on maternal neurobehavioral responses, and particularly those of sertraline, the most prescribed SSRI in United States (US) pregnancies. We administered C57Bl/6 females sertraline via a non-invasive, naturalistic approach (167 mg/L drinking water) from 2 weeks pre-conception through lactation (PND21) or for an equivalent duration in nonpregnant controls. We assessed behavior and molecular brain changes intrapartum and postpartum at ~1 year of age. Chronic sertraline reduced depressive- and anxiety-like behaviors. Pregnancy itself decreased anxiety-like and hedonic behaviors. RNA sequencing of maternal brain revealed only 52 differentially expressed genes (DEGs) in frontal cortex with sertraline. These DEGs over-represented functions related to immunity. In contrast, sertraline altered 962 targets in maternal hypothalamic paraventricular nucleus, with DEGs overrepresenting neurotransmission and neurodegeneration. We then discontinued sertraline and aged animals to approximately 1 year to test neurodegenerative phenotypes. Having one prior litter, regardless of peripartum sertraline, improved aged females' spatial learning and memory. Sertraline, regardless of postpartum status, improved working memory. Further, we found buffering of neurodegeneration-related gene network changes and increased excitatory synapse density in the hippocampus after peripartum sertraline. Peripartum sertraline alters maternal neurobiology and behavior in pregnancy and beyond, with long-term benefits to neurodegenerative processes. Pregnancy also exerts its own, lasting effects on learning and memory. These findings might be exploited in the future to abrogate neurodegenerative disease.

## Introduction

Perinatal mood and anxiety disorders (PMADs) impact approximately 1 in 5 pregnant and postpartum people [1, 2]. Rates have increased in recent years, and in particular since the COVID-19 pandemic era when PMAD prevalence more than doubled [3]. In 2022, mental health issues (suicide and overdose) were the leading cause of death in the first postpartum year [4] leading to the declaration of an Urgent Public Health Crisis in maternal health by The U.S. Department of Health and Human Services [5]. Selective serotonin reuptake inhibitors (SSRIs) are the most prescribed class of antidepressants, extensively utilized for their efficacy in treating mood disorders and PMADs [6]. SSRI use among people who are pregnant, postpartum, or lactating is common, with approximately 6–8% of pregnant people in the US prescribed an SSRI [7].

In response to the rising crisis of PMADs, antepartum (conception through delivery) SSRI use increased by nearly one-third over the early 2000’s in the US [8]. SSRIs are thought to modulate depressive symptoms by modifying serotonin uptake at the synapse. The serotonin transporter (SERT) takes up serotonin into the presynaptic neuron. SSRIs block SERT, preventing serotonin reuptake and allowing it to remain in the synaptic space where it continues to drive serotonin activity and thereby exerts therapeutic effects. Other SSRI mechanisms of action may also be involved in drug impacts. Recent work has revealed that neurotrophic, vascular, immunologic, microbiome, and other SSRI mechanisms are also involved [9–16]. Recent debates [17] have further emphasized a lack of clear understanding of SSRI mechanisms of action in the brain and periphery. Layered atop the dynamic neuroendocrine and metabolic changes of pregnancy, the neurobiological impacts of SSRIs in antepartum and postpartum become that much more unclear and in need of study [18].

The existent literature on SSRIs in peripartum has largely focused on impacts to offspring and potential teratogenicity [19–23]. Studies of peripartum SSRI often neglect to measure maternal biology (e.g., organ function, circulating drug levels, gestational outcomes), especially long-term, and are limited by confounds of intermittent blockade and administration stress (e.g. as with repetitive oral gavage or injection). These have their own, independent impacts on maternal-fetal biology and behavior, which are also related to serotonin-mediated phenotypes. For example, restraint stress alters mood-related phenotypes and is used to model depressive-like behavior and neurobiology in preclinical settings [24–26]. We address these limitations by maintaining a focus on maternal neurobiology and validating a noninvasive, naturalistic, chronic SSRI administration approach for use in pregnant mice: *Ad libitum* sertraline in drinking water throughout peripartum. While sertraline is the most prescribed SSRI used in US pregnancies [7], gestational sertraline is sparsely studied in preclinical models. Our approach non-invasively achieves a low dose of sertraline in murine pregnancy without impacts on gestational health to reveal effects on maternal brain and behavior in pregnancy and long-after, in the postpartum animal.

Our study goals were to examine the behavioral and molecular impacts of peripartum sertraline in a mouse model across cortical and subcortical brain regions involved in mood and stress response in both mice and humans [27]. We also explored gene expression related to neurodegeneration in young, pregnant animals after sertraline exposure. Subsequent, complementary behavioral studies in aged animals many months following a single pregnancy further examined learning and memory benefits of both pregnancy itself and of peripartum sertraline. Our work aimed to not only reveal immediate impacts of peripartum sertraline on brain health and function, but also to provide new insights into enduring consequences for maternal brain health across the lifespan.

## Methods and materials

Female C57Bl6/J mice (Jackson Labs, Bar Harbor, ME) approximately 6–8 weeks of age were administered sertraline (167 mg/L oral suspension; NorthstarRx LLC Pharmaceuticals) or water *ad libitum* via in-cage bottles starting two weeks prior to breeding then for the duration of pregnancy and lactation, or for an equivalent duration (approximately 8 weeks) in non-pregnant animals. For neurodegeneration studies, animals were aged to approximately one year (n = 3–4/condition) prior to behavior, molecular, and histology studies.

Plasma and amniotic fluid sertraline were confirmed by liquid chromatography–mass spectrometry/mass spectrometry (LC-MS/MS), and circulating serotonin was measured by ELISA. Mice were used for intrapartum behavioral assessment (n = 5–8/condition) by the forced swim test, tail suspension test, elevated plus maze, open field test, hedonic assessment (chocolate:chow preference testing), barnes maze, and radial arm maze.

**Fig. 1:**
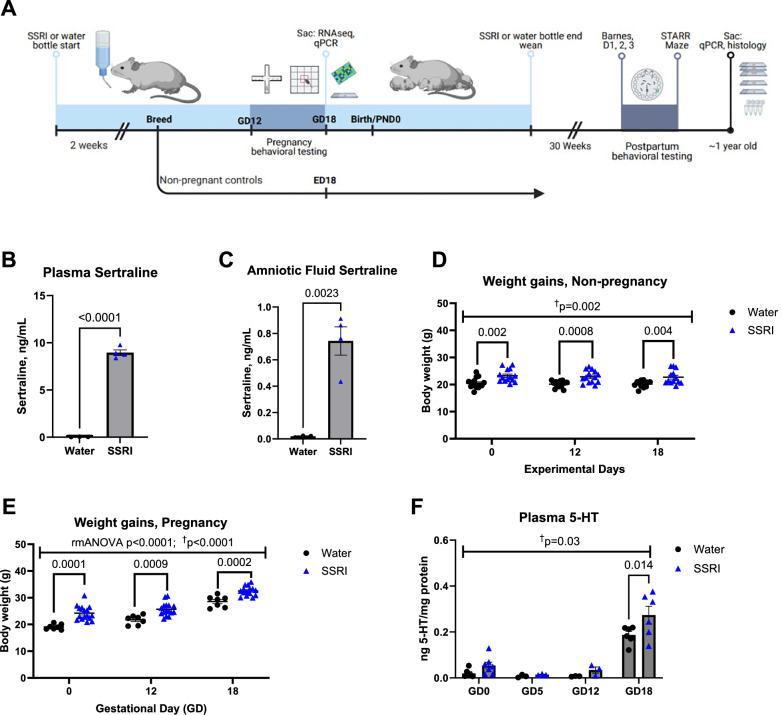
Validation of experimental approach to test effects of chronic oral sertraline in pregnancy. **A** Experimental timeline. Gestational day, GD; Experimental day, ED. **B** Oral sertraline (167 mg/L oral suspension) increases sertraline in plasma to approximately 9 ng/mL via LC-MS/MS and (**C**) in amniotic fluid after drinking water sertraline administration in pregnancy to approximately 0.7 ng/mL, both at GD18. Sertraline dosing began 2 weeks prior to breeding, and (**D,** **E**) weight gains were apparent after only 2 weeks of sertraline dosing (experimental day 0). Two weeks of sertraline pre-breeding increased weight of dams by GD or ED 0, which persisted across ED 12, 18 in nonpregnant animals (two-way repeated measures [R.M.] ANOVA main effect [M.E.] of sertraline: F[1,27] = 11.83, p = 0.002) and GD 12 and 18 in pregnant animals (two-way R.M. ANOVA M.E. of sertraline: F[1,21] = 29.60, p < 0.0001). **F** Plasma 5-HT is increased across pregnancy and in particular at GD18 by post-hoc Holm t-test (q = 0.014).

Tissues were collected by tissue punch from fresh frozen coronal brain sections for RNA sequencing (n = 3/condition/region; Illumina HiSeq platform) or qPCR studies of maternal brain (PVN and cortex) at gestational day 18 (GD18) (primers in Table 1). Complementary sections were immunostained for VGlut1 (1:1000 Millipore AB5905) and PSD-95 (1:200, Millipore AB9708) and synaptic density in the cortex, hippocampus, and corpus callosum quantified by ImageJ. Animal protocols received prior approval from the University of Iowa Institutional Animal Care and Use Committee (IACUC). Methods are fully detailed in the Supplement and outlined in Fig. 1A.

**Table 1:** Primers used in qPCR studies.

Target Gene	Sense/Forward	Antisense/Reverse
Psen1	CTCCTGCTCGCCATTTTCAAG	CACAAGGTAATCCGTGGCGA
Psen2	ACACTGAGAAGAACGGGCAG	AGGAGCATCAGGGAGGACAT
Gskip	GTTCGCCATTCCTTCACACG	GCAGCATTGAAGCCCTGTAAC
Frk	AGCAGGTCAGGAAGAAGCAC	CTCACCATACCTCCCGCTTC
Fyn	CAGCAAGACAAGGTGCGAAG	CCTGGGTATGGCACTCTTCC
Erk2	AAGACACAGCACCTCAGCAA	GTGTTCAGCAGGAGGTTGGA
Gsk3b	GGAGTGAAAAGCCAAGAGAACG	CAAAGGAGGTGGTTCTCGGT
Csnk1a1	CACAGGCAAGCAAACTGACAA	AACAAACGCTGCTCCAATCG
Csnk2a2	AAGGAGCCATTCTTCCACGG	TCCGTGAATGTTGTCCCAGG
Prkacb	CAAGAAAGGCAGCGAAGTGG	ATTACTCGGGGGAGGGTTCT
Mef2c	ATCCCGATGCAGACGATTCAG	AACAGCACACAATCTTTGCCT
Kalrn	CCGACTCTTGGACTACCTTATGA	CGACCCTACAGTTGTGCAG
Ptk2b	GCCCTTGAGCCGTGTAAAAGT	AGCTTGAAGTTCTTCCCTGGG
Stx1a	AAGATTGCCGAAAACGTGGAG	TGCTCAATGCTCTTTAGCTTGG
Synpo	TCCTCACCTAATGCCACACTC	GCTGGAGGGTTTTGGTTGATA
Dlgap	GCGGCAGCCTTATCTCCTTAG	GGATACGTGGTCACCCAACAT
Homer1	CCCTCTCTCATGCTAGTTCAGC	GCACAGCGTTTGCTTGACT
Atp2b1	CTCAGTTGCGTATAGCGGTGT	CAGCTCTGTAAGCGTAATTCCA
Mrc1	CTCTGTTCAGCTATTGGACGC	TGGCACTCCCAAACATAATTTGA
S100a8	AAATCACCATGCCCTCTACAAG	CCCACTTTTATCACCATCGCAA
Cdh23	GGAGGATTACCTACGGCTCAA	GTGTGGATCAGCTCGGAGA

## Results

### Chronic oral sertraline permeates maternal and amniotic biofluids and promotes weight gain without disrupting obstetric health

Our experimental timeline (Fig. 1A) involved breeding, behavioral testing, and maternal molecular and histologic brain studies at multiple timepoints: in gestation and 38 weeks later, when animals were approximately one year of age, equivalent to a mature adult human with reduced fertility [28]. Treatment of drinking water with sertraline did not change consumption across two days of testing (Day 1: *p* = 0.92; Day 2: p = 0.39) (Fig. S1, C, D). Given this consumption rate and sertraline concentration in the water bottles, daily consumption was calculated to approximate a 1.28 mg daily dose of sertraline, roughly equivalent to a 25–50 mg human dose after accounting for mouse and pregnancy metabolism and drug clearance differences [29] (using animal equivalent dose calculation based on body surface area, as described previously [30, 31]: Km ratio of mouse/human= 3/37 × 25–50 mg daily dose= 2/3–4.05 mg/day x 0.5 pregnancy clearance adjustment= 1.015–2.025 mg/day). We further found that naturalistic administration of sertraline (167 mg/L oral suspension) *ad libitum* via in-cage drinking water throughout pregnancy achieves plasma sertraline levels at GD18 of approximately 9 ng/mL (Control: 0.038 ± 0.01 ng/mL, n = 3; SSRI: 8.952 ± 0.29, n = 4; p < 0.0001; Fig. 1B) and elevated amniotic fluid sertraline (Control: 0.020 ± 0.004 ng/mL, n = 3; SSRI: 0.74 ± 0.11, n = 4; p = 0.0023; Fig. 1C) via LC-MS/MS (Fig. S1A, B).

Two weeks of sertraline pre-breeding increased weight of dams by GD or ED 0, which persisted across ED 12, 18 in nonpregnant animals (two-way repeated measures [R.M.] ANOVA main effect [M.E.] of sertraline: F[1,27] = 11.83, p = 0.002; Fig. 1D) and GD 12 and 18 in pregnant animals (two-way R.M. ANOVA M.E. of sertraline: F[1,21] = 29.60, p < 0.0001; Fig. 1E). There were no differences in baseline animal weight by group (pregnant sertraline vs water: p = 0.54; non-pregnant sertraline vs water: p = 0.12; n = 11–21/group), nor weight gain interactions between sertraline treatment and pregnancy status (p = 0.44; Fig. 1D, E). Two weeks of sertraline pre-breeding significantly increased plasma 5-HT concentrations in sertraline treated animals, driven by GD18 (two-way ANOVA M.E of sertraline: F[1, 29] = 5.467, *p* = 0.027; post-hoc *q* = 0.014; Fig. 1F). Sertraline did not alter maternal GD16 urine protein (Fig. S1E), litter size (Fig. S1F), nor GD or ED 16–18 systolic blood pressure (Fig. S1G).

### Pregnancy and sertraline exert differential effects on behavior

**Fig. 2:**
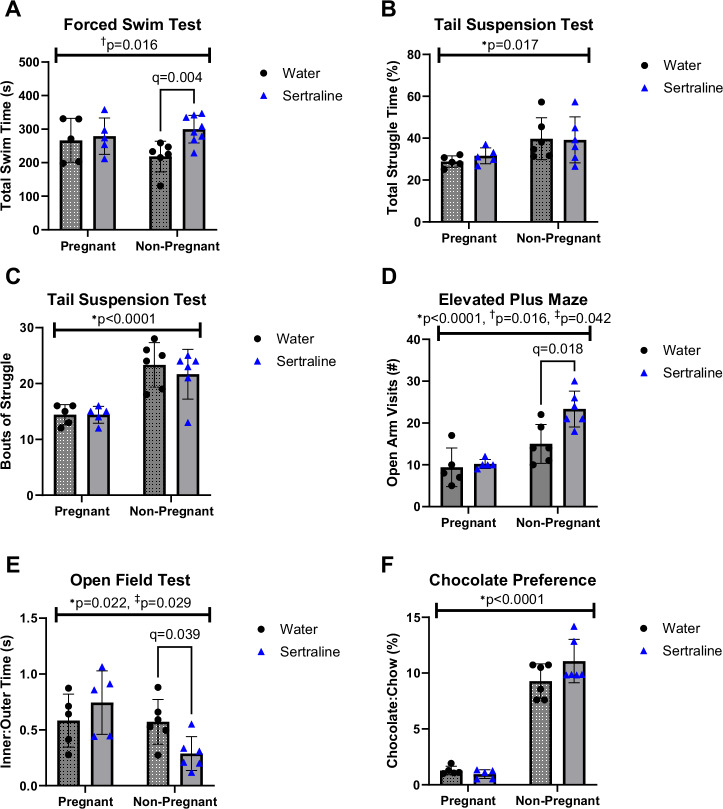
Non-pregnant and pregnant female behavior after chronic, oral sertraline. **A** On the Forced Swim Test (FST), sertraline exposure significantly increased total swim time driven by non-pregnant animals (two-way ANOVA main effect (M.E) of SSRI: F[1, 20] = 5.017, †*p* = 0.037; post-hoc difference by two-step Bejamini, Krieger, and Yekutieli (BKY) method in non-pregnant animals, *q* = 0.004). **B** Total struggle time in the Tail Suspension Test (TST) was increased in non-pregnant animals only (two-way ANOVA M.E of pregnancy: F[1, 18] = 7.007, *p = 0.017) and (**C**) TST bouts of struggle were significantly increased in non-pregnant animals only (two-way ANOVA M.E of pregnancy, F[1, 18] = 32.04, *p < 0.0001). **D** Pregnant mice exhibited reduced open arm visits on the Elevated Plus Maze (EPM), driven by non-pregnant sertraline-treated animals (two-way ANOVA M.E of pregnancy: F[1, 18] = 29.77, *p < 0.0001; *p*ost-hoc difference by BKY method, *q* = 0.018), sertraline-treated animals had significantly increased open arm visits, driven by the non-pregnant group (two-way ANOVA M.E of sertraline: F[1, 18] = 7.077, †*p* = 0.0159, post-hoc difference by two-step BKY method, *q* = 0.018), and sertraline treatment increased open arm visits in the non-pregnant group only (ANOVA pregnancy x sertraline interaction: F[1, 18] = 4.814, ‡*p* = 0.0416). **E** Non-pregnant animals exhibited significantly less inner:outer time on the open field test, which was driven by non-pregnant animals (two-way ANOVA M.E of pregnancy: F[1, 18] = 6.218, *p = 0.022; post-hoc difference by two-step BKY method, *q* = 0.039). **F** Preference for a high**-**sugar treat (chocolate kisses) versus normal chow was increased in non-pregnant animals relative to pregnant animals regardless of sertraline status (two-way ANOVA M.E of pregnancy: F[1, 18] = 252.1, *p < 0.0001). † Main effect of sertraline by two-way ANOVA; * main effect of pregnancy by two-way ANOVA; ‡ Pregnancy-by-sertraline interaction by two-way ANOVA.

To further test our model of naturalistic sertraline administration for effects on mood, anxiety-like, and hedonic behaviors and for interactions with pregnancy, we assessed behavior in late pregnancy and in non-pregnant controls. The forced swim test revealed that sertraline increased swim time, an effect driven by the non-pregnant group (two-way ANOVA M.E. of sertraline: F[1, 20] = 5.017, *p* = 0.016 post-hoc difference by two-step Benjamini, Krieger, and Yekutieli (BKY) method in non-pregnant animals, *q* = 0.004; Fig. 2A).

The tail suspension test revealed main effects of pregnancy: significantly less struggle time (two-way ANOVA M.E. of pregnancy: F[1, 18] = 7.007, *p* = 0.017; Fig. 2B) and fewer bouts of struggle among pregnant mice (F[1, 18] = 32.04, *p* < 0.0001; Fig. 2C).

On the elevated plus maze (EPM), pregnant animals made fewer visits to the open arms (two-way ANOVA pregnancy M.E.: F[1, 18] = 29.77, *p* < 0.0001; post-hoc *q* = 0.018; Fig. 2D) and sertraline animals made significantly more open arm visits, driven by the non-pregnant group (two-way ANOVA M.E. of sertraline: F[1, 18] = 7.077, *p* = 0.016, post-hoc *q* = 0.018; Fig. 2D).

On the open field test (OFT), non-pregnant animals spent significantly less time in the inner region of the OFT relative to the outer region (two-way ANOVA M.E. of pregnancy: F[1, 18] = 6.218, *p* = 0.022; Fig. 2E). Sertraline exposure interacted with pregnancy, decreasing time spent in the inner relative to outer region in non-pregnant animals only (interaction: F[1, 18] = 5.593, *p* = 0.024; post-hoc *q* = 0.039; Fig. 2E). There were no significant differences in total distance traveled in the OFT apparatus, regardless of pregnancy or sertraline status (Fig. S1H).

Hedonic behavior was assessed by measuring preference for a high-sugar treat (chocolate) versus standard chow. This unique alternative to the sucrose preference test allowed for continuous sertraline administration via in-cage water bottle. Non-pregnant animals, regardless of sertraline, consumed more chocolate vs chow compared to pregnant counterparts (two-way ANOVA M.E. of pregnancy: F[1, 18] = 252.1, *p* < 0.0001; Fig. 2F). Total chow or chocolate treat consumption revealed no change by sertraline status (Fig. S1I, J). Pregnancy suppressed appetite for both chow (two-way ANOVA main effect of pregnancy: F[1, 14] = 17.14; *p* = 0.001) and chocolate (F[1, 14] = 138.0; *p* < 0.0001) over 24 h in late gestation (Fig. S1I, J).

Collectively, these results demonstrate that sertraline reduced depressive- and anxiety-like behaviors, as measured by performance on the FST and EPM, respectively. Pregnancy obscured effects and drove main effects on the hedonic assay of chocolate consumption, TST, EPM, and OFT, where it interacted with sertraline status to decrease inner:outer time.

### Transcriptomics of late-gestation maternal brain paraventricular nucleus and frontal cortex reveal neurodegeneration and neuroimmune changes, respectively, due to sertraline

We next tested whether sertraline led to cortical and subcortical changes in maternal brain. We compared sertraline to water-treated maternal brain transcriptomics from the last day of gestation (GD 18) in frontal cortex and in the hypothalamic paraventricular nucleus (PVN) (N = 3/condition/region). These brain regions were selected for their critical roles in the regulation and generation of affective and mood-related phenotypes [32]. Nonpregnant animal transcriptional changes with SSRI exposure have been described previously [33] and were beyond the scope of the present study, given our focus on neurobiology *within* pregnancy.

**Fig. 3:**
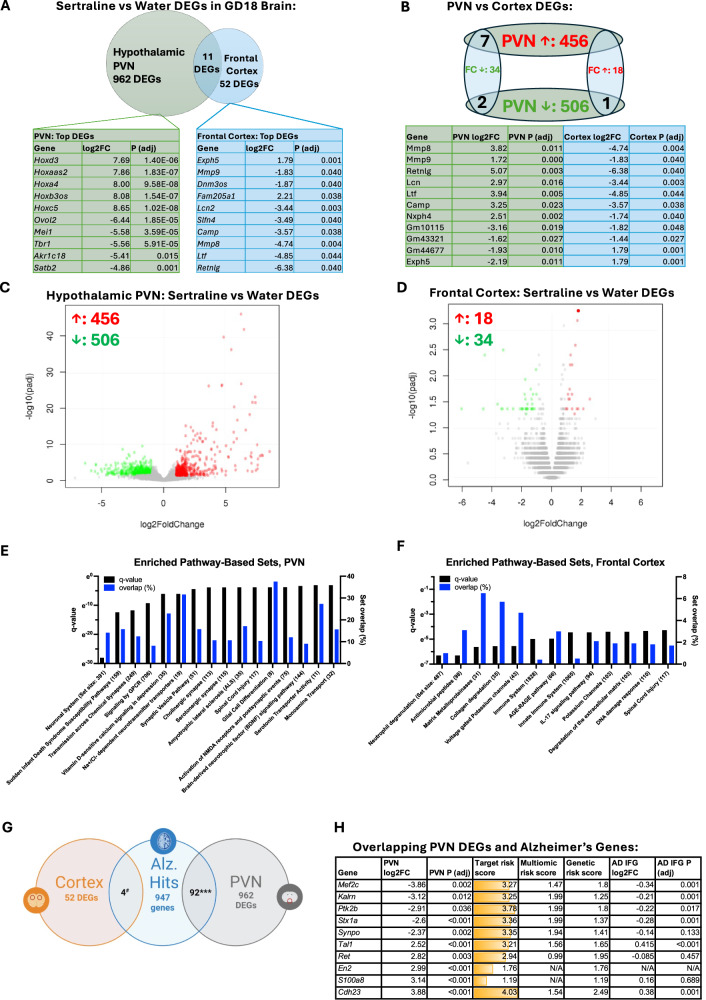
Differential gene expression in pregnant dam frontal cortex and paraventricular nucleus following peripartum treatment with sertraline. **A** Differentially expressed genes (DEG) in RNA sequencing datasets from hypothalamic paraventricular nucleus (PVN) and frontal cortex reveal changes in each region and overlapping changes. Top 10 DEGs with characterized protein products ranked by fold change (FC) with associated adjusted (adj) *p* values listed. PVN list contains top 5 up-regulated and down-regulated, by FC, respectively. Cortex includes top 10 by absolute FC. **B** Overlapping DEGs between PVN and frontal cortex datasets. **C**) Volcano plot visualization of global transcriptional changes in the PVN, with each data point representing a gene. DEGs with an adjusted *p*-value < 0.05 and a log2FC > 1 are indicated by red; log2FC < −1 indicated by green dots. **D** Volcano plot of DEGs in frontal cortex. **E** Select enriched pathway-based sets that are significantly over-represented among DEGs in PVN and (**F**) Cortex. Pathways are sourced from Reactome, Wikipathways, and KEGG via the Consensus Pathway Database (CPDB; Max Planck Institute). Details of pathway lists are provided in Tables 2, 3. **G** Significant overlap between PVN DEGs and Alzheimer’s Disease (AD) target genes, identified by the National Institute on Aging’s Accelerating Medicines Partnership in Alzheimer’s Disease (AMP-AD) consortium *** *P* < 0.0001 by chi-square. Cortical DEGs did not significantly overlap with Alzheimer’s (Alz.) genes. #P = 0.061 by chi-square. Created in BioRender. Gumusoglu, S. (2024) BioRender.com/d71t292. **H** Overlap between PVN DEGs and AD target genes included 92 genes; the top 5 most up-regulated and top 5 most down-regulated are listed by FC and detail target risk score (sum of multiomic and genetic risk scores, range 0–5), multiomic risk score (summary score of transcriptomic and proteomic studies supporting association of target gene with late-onset AD, range 0–2), and genetic risk score (summary score of genetic evidence supporting target gene’s association with late-onset AD drawn from genetic and phenotypic evidence from human and model organism studies, range 0–3). Target risk scores are gradient filled by magnitude, with darkest yellow being the highest score. Inferior frontal gyrus (IFG) expression in AD FC and P values are provided for comparison. AD scores and values all from https://agora.adknowledgeportal.org/.

Differentially expressed gene (DEG) analyses revealed 506 down-regulated and 456 up-regulated genes in the sertraline-exposed maternal PVN and 34 down-regulated and 18 up-regulated genes in sertraline-exposed maternal frontal cortex (Fig. 3C, D; Supplementary Tables 1, 2; PCA: Fig. S6A, B). Of these, 11 were overlapping between regions, though only two in valence (downregulated in both; Fig. 3B). DEG enrichment for pathway-based gene sets was tested and revealed over-representation of neuronal and synaptic sets (e.g., Neuronal System, Transmission across Chemical Synapses, Synaptic Vesicle Pathway) in PVN and immunologic sets (e.g., Neutrophil degranulation, Immune System, IL-17 signaling pathway) in frontal cortex with sertraline (Fig. 3E, F; Tables 2, 3). Among the drivers of these changes were DEGs including synaptic genes *Satb2* and *Tbr1* in PVN and immune regulatory genes *Mmp9* and *Mmp8* in frontal cortex.

**Table 2:** Enrichment of gene ontology pathway-based sets from cortical DEGs curated via the Consensus Pathway Database (CPDB; Max Planck Institute).

Pathway name	Set size	Candidates contained (%)	q-value	p-value	Pathway source
Neutrophil degranulation	487	5 (1.0%)	0.000135	0.00182	Reactome
Antimicrobial peptides	98	3 (3.1%)	0.000161	0.00182	Reactome
Matrix Metalloproteinases	31	2 (6.5%)	0.000531	0.00361	Wikipathways
Collagen degradation	35	2 (5.7%)	0.000678	0.00384	Reactome
Voltage gated Potassium channels	43	2 (4.7%)	0.00102	0.00387	Reactome
Immune System	1828	7 (0.4%)	0.00201	0.00684	Reactome
AGE-RAGE pathway	66	2 (3.0%)	0.0024	0.00699	Wikipathways
Innate Immune System	1065	5 (0.5%)	0.00473	0.0116	Reactome
IL-17 signaling pathway - Homo sapiens (human)	94	2 (2.1%)	0.00479	0.0116	KEGG
Potassium Channels	103	2 (1.9%)	0.00573	0.0122	Reactome
Degradation of the extracellular matrix	103	2 (1.9%)	0.00573	0.0122	Reactome
DNA damage response (only ATM dependent)	110	2 (1.8%)	0.00651	0.013	Wikipathways
Spinal Cord Injury	117	2 (1.7%)	0.00733	0.0139	Wikipathways

**Table 3:** Enrichment of gene ontology pathway-based sets from PVN DEGs curated via the Consensus Pathway Database (CPDB; Max Planck Institute).

Pathway name	Set size	Candidates contained (%)	q-value	p-value	Pathway source
Neuronal System	391	55 (14.1%)	4.76E-16	6.67E-13	Reactome
Sudden Infant Death Syndrome (SIDS) Susceptibility Pathways	159	25 (15.7%)	5.90E-09	4.13E-06	Wikipathways
Transmission across Chemical Synapses	249	31 (12.4%)	2.84E-08	7.97E-06	Reactome
Signaling by GPCR	706	57 (8.1%)	5.33E-07	9.35E-05	Reactome
Vitamin D-sensitive calcium signaling in depression	35	8 (22.9%)	6.38E-05	0.00229	Wikipathways
Na + /Cl- dependent neurotransmitter transporters	19	6 (31.6%)	7.64E-05	0.00229	Reactome
Synaptic Vesicle Pathway	51	8 (15.7%)	0.00097	0.01187	Wikipathways
Cholinergic synapse - Homo sapiens (human)	113	12 (10.6%)	0.00215	0.02097	KEGG
Serotonergic synapse - Homo sapiens (human)	115	12 (10.6%)	0.00215	0.02097	KEGG
Amyotrophic lateral sclerosis (ALS)	35	6 (17.1%)	0.00263	0.02203	Wikipathways
Spinal Cord Injury	117	12 (10.3%)	0.00289	0.02203	Wikipathways
Glial Cell Differentiation	8	3 (37.5%)	0.00323	0.02227	Wikipathways
Activation of NMDA receptors and postsynaptic events	75	9 (12.0%)	0.00331	0.02271	Reactome
Brain-derived neurotrophic factor (BDNF) signaling pathway	144	13 (9.0%)	0.00592	0.03418	Wikipathways
Serotonin Transporter Activity	11	3 (27.3%)	0.00867	0.04405	Wikipathways
Monoamine Transport	32	5 (15.6%)	0.0089	0.04504	Wikipathways

PVN DEGs implicated in Alzheimer’s Disease (AD) were among the top hits, for example *Synpo* and *Mef2c* [34–40]. Significant overlap between PVN DEGs and AD genes more broadly was tested by over-representation analyses against the National Institute on Aging’s Accelerating Medicines Partnership in Alzheimer’s Disease (AMP-AD) consortium dataset (agora.adknowledgeporta.org). Cortical DEGs did not significantly overlap with AD genes (P = 0.061 by chi-square) but overlap between PVN DEGs and AD genes was significant (P < 0.0001 by chi-square) and included 92 DEGs (Fig. 3G). AD target risk scores of overlapping DEGs ranged from 1.19 (*S100a8)* to 4.03 (*Cdh23)*, indicating strong genetic and phenotypic evidence of association with AD (Fig. 3H).

To examine specificity for AD versus depression genes, which might also be altered by chronic SSRI use, we also evaluated overlap with major depressive disorder (MDD) variants identified previously in three large genome-wide association studies [41–43]. No cortical DEGs and only two PVN DEGs were found to overlap with MDD intron variants: rs9530139 (mapped to *B3GLCT*) and rs112348907 (mapped to *KCNQ5*), both identified in a large study of loci associated with MDD in individuals of European descent [42, 44]. We further evaluated overlap with DEGs identified by transcriptomic profiling of 87 cerebral cortical samples from subjects with MDD (FDR < 0.05) [44]. Of these DEGs, none overlapped with cortical RNA seq hits but 11 overlapped with PVN RNA seq DEGs (see Supplementary Table 2, “Overlap with PMID: 29439242; FDR < 0.05” column). PVN DEGs did not significantly over-represent MDD DEGs from this dataset (P = 0.89 by chi-square).

### Both postpartum status and perinatal sertraline improve learning and memory with age

Given our transcriptomic findings linking sertraline in pregnancy with AD-associated gene expression intrapartum, we additionally tested learning and memory behaviors after aging mice to approximately 1 year. We utilized a three-day Barnes maze and a one-day radial arm maze task [45, 46] to assess spatial learning/memory and working memory, respectively.

**Fig. 4:**
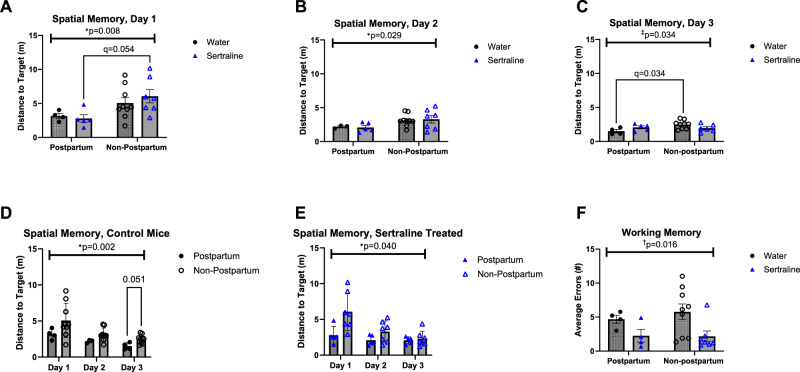
Learning and memory in aged animals was altered by peripartum sertraline and by reproductive history. Barnes Maze was used to assess spatial memory in aged animals. **A** On the Barnes Maze, postpartum status significantly improved performance measured by distance to the target on day one (two-way ANOVA main effect (M.E) of pregnancy: F[1, 21] = 8.661, *p = 0.008) and (**B**) on day 2 (two-way ANOVA M.E of pregnancy: F[1, 20] = 5.485, *p = 0.029). **C** On day 3 of Barnes maze, postpartum status improved performance in control animals, but not sertraline-treated animals (ANOVA Interaction: F[1, 19] = 4.203, ‡*p* = 0.034; post-hoc difference by two-step Benjamin, Krieger, and Yekutieli (BKY) method, *q* = 0.034). **D** Control mice demonstrated that postpartum animals had significantly improved performance relative to non-postpartum animals(two-way ANOVA M.E of pregnancy: F[1, 11] = 12.92, *p = 0.004; post-hoc trend difference by two-step BKY method, *q* = 0.051). **E** Postpartum status was also sufficient to improve performance in sertraline treated mice (two-way ANOVA M.E of pregnancy: F[1, 10] = 5.244, *p = 0.045). **F** STARR Maze was used to assess working memory in aged animals. Sertraline treatment significantly reduced working memory errors, regardless of pregnancy status (two-way ANOVA M.E of sertraline: F[1, 20] = 9.094, †*p* = 0.016). † Main effect of sertraline by two-way ANOVA; * main effect of pregnancy by two-way ANOVA; ‡ Pregnancy-by-sertraline interaction by two-way ANOVA.

On day 1 of Barnes maze testing, there was no difference in overall baseline locomotion in either postpartum (trial 1, day 1: Water 2.677 + /− 2.051, SSRI 2.766 +/− 2.232, *p* = 0.952) or nonpostpartum animals (trial 1, day 1: Water 5.177 +/− 5.552, SSRI 9.082 +/− 6.024, *p* = 0.199). Postpartum status improved spatial learning and memory performance on day 1 (two-way ANOVA M.E of pregnancy: F[1, 21] = 8.661, *p* = 0.008; Fig. 4A) and day 2 (F[1, 20] = 5.485, *p* = 0.029; Fig. 4B). On day 3, postpartum animals had improved performance in control animals but not sertraline-treated animals (ANOVA interaction: F[1, 19] = 4.203, *p* = 0.034; post-hoc *q* = 0.034; Fig. 4C).

We next analyzed Barnes maze performance to understand postpartum impacts across test days. Among water controls, postpartum aged mice demonstrated significantly improved performance over all three days of testing (two-way ANOVA M.E. of pregnancy: F[1, 11] = 5.409, *p* = 0.002; day 3 post-hoc *q* = 0.051; Fig. 4D). Similarly, postpartum sertraline-treated animals demonstrated improved performance on the Barnes Maze over all three days of testing (F[1, 10] = 5.244, *p* = 0.040; Fig. 4E). No single day of testing drove these changes by post-hoc test.

Working memory was next assessed via a one-day radial arm maze test. Sertraline-treated females exhibited fewer errors on this task than controls, regardless of postpartum status (two-way ANOVA M.E. of sertraline: F[1, 20] = 9.094, *p* = 0.016; Fig. 4F).

### Hippocampal and cortical neurodegeneration-related molecular phenotypes are buffered by peripartum sertraline and by postpartum status

**Fig. 5:**
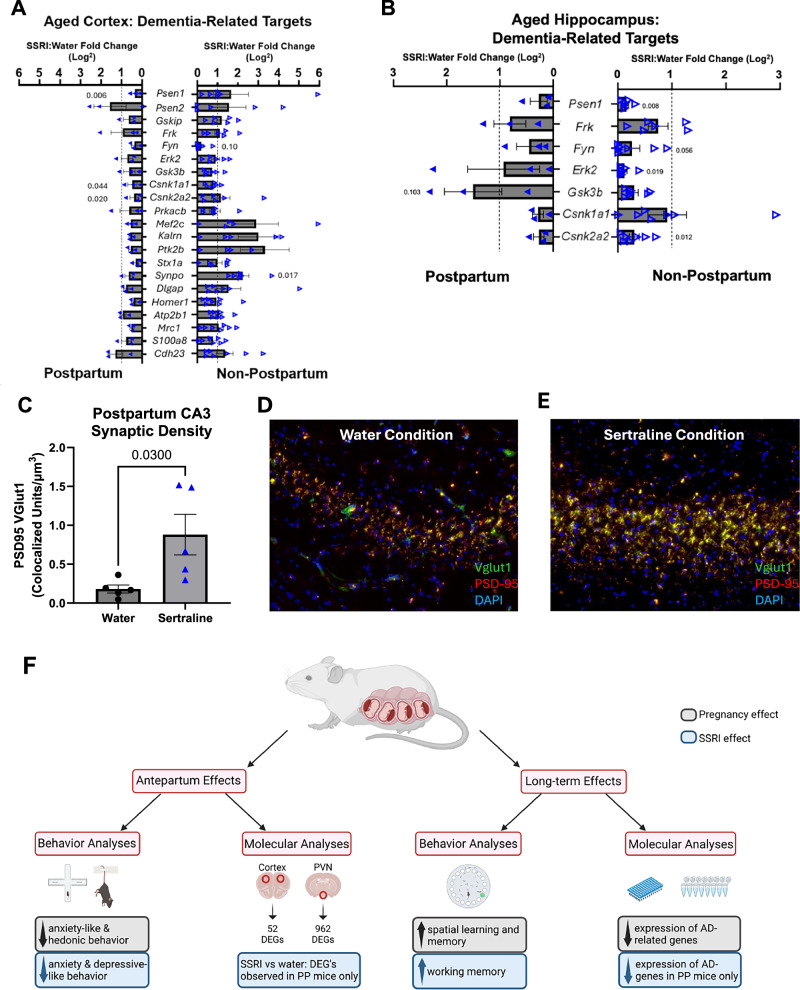
Cortical and hippocampal changes in postpartum dams suggest buffering of neurodegeneration-related pathways by sertraline and by reproductive history. **A** In postpartum aged cortex, there was significantly decreased expression of *Psen1* (two-tailed, unpaired t-test, *p* = 0.006), *Csnk1a1* (*p* = 0.044), and *Csnk2a2* (*p* = 0.020*)* with sertraline. In non-postpartum animals, there was trend-decreased *Fyn* expression (*p* = 0.10), and significantly increased *Synpo* expression (*p* = 0.017) compared to water-treated non-postpartum controls. **B** Analyses of aged hippocampus via qPCR revealed no significant differences in sertraline-treated postpartum animals, but decreased expression of *Psen1* (two-tailed, unpaired t-test, *p* = 0.008), *Fyn* (*p* = 0.056), *Erk2* (*p* = 0.019), and *Csnk2a2* (*p* = 0.012) in non-postpartum sertraline-treated animals. **C** In postpartum, there were significantly increased colocalized Vglut1 and PSD-95 synaptic puncta in hippocampal CA3 among aged, sertraline-treated (vs water control) dams (two-tailed t-test, *p* = 0.030). **D** Representative images at 20x used to quantify CA3 synaptic density for water-treated animals **E** and sertraline-treated animals. Orange color indicates Vglut1 and PSD-95 overlap at a synapse. **F** Summary of main antepartum and long-term effects on neurobehavior of peripartum SSRI and/or pregnancy itself; postpartum (PP); Alzheimer’s Disease (AD). Created in BioRender. Gumusoglu, S. (2024) BioRender.com/d71t292.

Given buffering of learning and memory behaviors by sertraline and postpartum status, we next examined long-term molecular changes in brain regions subserving these functions in our aged animal cohort: the frontal cortex and hippocampus. Interaction analyses of DEGs from GD18 maternal brain RNAseq identified targets: RNAseq DEGs *Grin2a*, *Grin2b*, *Itpr1*, and *Plcb1* strongly interact with *Gsk3b*, *Psen1*, and others in the “Alzheimer’s disease” (WikiPathways) network (Fig. S2). qPCR revealed disruption of genes by peripartum sertraline in this network in aged, postpartum cortex, including significantly decreased cortical P*sen1* (*p* = 0.006), *Csnk1a1* (p = 0.044), and *Csnk2a2* (*p* = 0.020) (Fig. 5A). Contrary to this, only *Synpo* (*p* = 0.017) was significantly decreased by peripartum sertraline in aged, non-postpartum cortex (Fig. 5A). Also in aged mice, qPCR of postpartum hippocampus revealed no significant differences in gene expression with sertraline, while in non-postpartum animals’ expression of AD-related *Psen1* (*p* = 0.008), *Erk2* (*p* = 0.019), *Fyn* (*p* = 0.056), and *Csnk2a2* (*p* = 0.012) were decreased or trend-decreased compared to water controls (Fig. 5B).

Complementary studies in cortex and hippocampus evaluated whether AD network genes were also changed by chronic SSRI in late gestation (GD 18) or in non-pregnant females. Overall, we found more wide-spread changes in AD-related gene expression in late gestation than in aged brain; 8 genes significantly changed in GD 18 cortex by SSRI and 4 in nonpregnant cortex by SSRI (Fig. S5A). As in aged brain, hippocampal changes were subtle, with only *Synpo (p* = 0.017*)* and *Csnk1a1 (p* = 0.006*)* significantly decreased (Fig. S5B), as they were in aged, postpartum cortex (Fig. 5A).

AD genes shown to be differentially impacted by peripartum sertraline in postpartum vs non-postpartum females were also correlated with learning and memory behaviors. While there was no main effect of sertraline on day 3 Barnes maze performance (Fig. 4C), distance to the target on the final day of Barnes maze testing, day 3, when maximal learning has occurred, was significantly and negatively correlated with cortical *Psen1* (linear regression, r^2^ = 0.598, *p* = 0.041; Fig. S4A) and *Csnk2a2* (r^2^ = 0.743, *p* = 0.013; Fig. S4B), but not with *Csnk1a1* expression (r^2^ = 0.419, *p* = 0.116; Fig S4C). These protein regulatory genes were significantly down-regulated in postpartum cortex with peripartum sertraline. No correlations were significant in non-postpartum mice (Fig. S4D–F), demonstrating a specific effect of peripartum sertraline in aged postpartum mice only.

In aged postpartum sertraline-treated animals, there was a significantly increased density of colocalized Vglut1 and PSD-95 double-positive excitatory synaptic puncta in hippocampal CA3 (*p* = 0.030; Fig. 5C–E). Volumetry studies of cortex, hippocampus including CA regions and dentate gyrus, and corpus callosum revealed no changes by pregnancy nor sertraline status (Fig. S3). Collectively, these results demonstrate buffering of AD-related changes by sertraline within postpartum animals only; this is evident in brain regions critical to learning and memory processes.

## Discussion

While selective serotonin reuptake inhibitors (SSRIs) are widely prescribed during pregnancy, the literature on their acute and long-term impacts on intrapartum neurobiology remains limited. Here, we utilized a non-invasive, naturalistic preclinical model to investigate the consequences of chronic perinatal sertraline on maternal behavior, neurobiology, and neurodegeneration-associated phenotypes across the lifespan. We describe novel insights into neuroprotection by peripartum SSRIs and by pregnancy itself and reveal potential underlying molecular mechanisms relevant to female neurobiology across the lifespan.

We tested whether chronic, oral delivery of sertraline to mice achieves maternal neurobiological changes without obstetric disruptions. This approach avoids the chronic stress confounds of oral gavage [47] or repetitive injections [48], does not disrupt obstetric health, and achieves clinically-relevant levels of sertraline [30, 31] without major side-effects, as reported with clinical use of extended-release antidepressants [49].

Our behavior results indicate reduced sertraline modification of behavior in late gestation. However, pregnancy itself had significant impacts on locomotion (OFT), mood/anhedonia (TST, chocolate preference), and anxiety-like behaviors (EPM). Importantly, this is not simply a result of reduced activity, as demonstrated by animal locomotor behavior on the open field. Changes may be due to maternal brain biology, pharmacokinetics which reduce sertraline efficacy, an increased volume of distribution, or metabolism changes due to increased estrogen. For example, sertraline metabolism and clearance are increased from the second to third trimesters of pregnancy [29] as a function of hepatic cytochrome P450 activity, which varies greatly between individuals [50]. Our intrapartum behavior results support the conclusion that, as others have argued, SSRI dose adjustment should be considered to offset drug turnover and maintain pharmacotherapy in pregnancy [51, 52]. Sertraline, and indeed SSRI’s more broadly, have increased drug metabolism across pregnancy [51, 53] (as much as 143%) [29]. Furthermore, 50 mg/day represents only a starting, non-therapeutic dose in many cases. Future studies will test increased doses, as well as drug metabolism in this model across murine pregnancy and across periphery and brain. This is necessary to advance a translational understanding of drug impacts in peripartum, and to optimize the utility of animal models in pregnancy pharmacology.

Despite blunted changes to behavior in pregnancy, sertraline significantly changed cortical and hypothalamic gene expression in late pregnancy. To our knowledge, this is the first report of transcriptomic changes in late-gestation maternal brain after intrapartum sertraline. Changes were greater in the hypothalamic paraventricular nucleus (PVN) relative to frontal cortex. Prior reports have similarly described more pronounced PVN gene expression changes compared to the frontal cortex with SSRI use in non-pregnant animals [54]. Changes are not driven by SSRI-induced histone acetylation, implicating an alternate regulatory mechanism [55]. Hypothalamic cells actively sample cerebrospinal fluid and play a key role in environmental and stress responses [56]. Circulating immune, metabolic, steroidal, and other factors that are changed in depression and with SSRI use may act directly on regulatory elements to modify hypothalamic gene expression [57, 58]. For example, glucocorticoids directly repress cAM-stimulated CRF promotor activity in hypothalamic cells [59]. The neuroanatomy of the PVN supports its role in endocrine signaling and sensing of the periphery—retrograde signaling via posterior pituitary axons [60]; vagus-to-nucleus of the solitary tract (NTS) circuits [61]; and subfornical organ (SFO) and organum vasculosum of the lamina terminalis (OVLT) projections all enable peripheral neuroendocrine signaling to the PVN [62], which can drive central metabolic and transcriptional changes. Studies of these mechanisms in pregnancy, when blood volume and cerebrospinal fluid metabolites are significantly altered, remain absent, however.

Prior work has shown that SSRI use alters expression of AD-related genes [55]. Our work expands on this by including a pregnant versus non-pregnant comparison and, perhaps most unexpectedly, demonstrates that both postpartum status *and* perinatal sertraline may modify AD-relevant gene expression. We observed decreased expression of neurodegeneration-related genes (Fig. 5A) in cortex of aged postpartum dams after SSRI administration, and of GD 18 but not non-pregnant dams after SSRI administration (Fig. S5). Central nodes of AD gene interaction networks including cortical *Psen1* and *Csnk2a2* are down-regulated in postpartum relative to non-postpartum mice by peripartum sertraline and are inversely correlated with learning and memory performance. While we did find this interesting correlation, we not find a main effect of sertraline itself on day 3 maze performance. Given high variance in behavior, it is possible that we were under-powered to detect such a difference and future replication work will expand the cohort size. *Psen1* mutation status has been shown to modify postpartum cognitive deficits in humans, which are correlated with immunosuppressive changes [63]. Our RNAseq studies further demonstrate immunologic changes in the cortex with peripartum sertraline use, in particular invoking IL-17 pathway dysregulation. *Psen1* mutation status has been shown to regulate Th17 T cell effector responses and T cell differentiation via γ-secretase signals [64]. This mechanism of *Psen1* neuroimmune modulation via IL-17 cascades may thereby underlie neuroimmune phenotypes driving some AD pathology. The immunologically dynamic events of pregnancy may further serve as a critical substrate for initiation of these processes [65], which requires additional investigation.

Beyond inflammation, our work implicates additional mechanisms of peripartum sertraline impacts on the ageing brain. SSRIs modulate neuroplasticity [66], synaptic function [67], and neurogenesis [68], which are crucial for learning and memory processes. Indeed, we find transcriptomic changes in neuroplasticity and synaptic genes after gestational sertraline administration, including to *Plk2* [69], *Nsmf* [70], *Shank2* [71], *Shank3* [72]*, Mef2C* [73], *Grin2A* [74], and others. Synaptic remodeling in pregnancy has been shown previously in both human and animal studies [75–77] and may be a critical mechanism for neuroprotection. Future work will need to sequence additional cohorts of mice and expand statistical power to validate these DEGs and identify additional ones. Greater power will also allow examination of DEGs and the functional pathways involved by valence, which will provide additional insights.

While parity and SSRI have both previously been associated with reduced neurodegeneration [78, 79], driving mechanisms remain unclear; our results offer a valuable first view. We confirm previous findings: SSRI’s may modify neurodegenerative mechanisms at the molecular and behavioral levels, implicating neuroimmune [80], plasticity [81], and synaptic processes [82]. Unlike this prior work, however, we explore these dynamics within a pregnancy and postpartum context. To our knowledge, this is the first study to examine pregnancy and postpartum changes in neurodegenerative pathways as they relate to chronic peripartum sertraline.

Future work is needed to further validate our study findings and expand them to explore SSRI rescue in depression-like models. While daily handling and behavioral assessments (especially tail suspension and forced swim) [83] have been shown to induce depression-like changes in murine models [84–86], we did not employ a traditional depression induction model concurrent with sertraline here. It will be important for future work to test the interactions of sertraline-induced brain and behavior changes we report with depression itself, and for extension of this work to non-pregnant animals to reveal pregnancy specificity of all molecular impacts (RNA sequencing was only done at GD 18 here). For example, our prior work in a chronic restraint model, which leads to depression-like changes in mice [87], implicates pro-inflammatory changes in dams [88]. These inflammatory changes may be modified by chronic sertraline administration, as we have suggested occurs in other pro-inflammatory conditions of pregnancy such as preeclampsia [11, 89, 90]. Our planned and ongoing studies will evaluate this potential given the promise of results demonstrated here.

As clinicians, the NIH, and others have begun to advocate with increasing fervor, the best way to enhance health in pregnancy is to “protect pregnant people through research not from research” [91]. Concerns about teratogenicity and other risks have restrained use and study of SSRIs in pregnancy, as discussed previously [92]. Despite the risks of untreated depression, multinational research finds that 75.4% of women discontinue antidepressants during pregnancy due to health concerns [93, 94]. Human cohort studies struggle to directly address these concerns; the causal, neurobiological effects of antidepressants in human pregnancy are difficult to ascertain due to study confounds and experimental and ethical limitations precluding randomized controlled trials. Observational studies are confounded by differences in co-medications, dosing, comorbidities and symptom severity, healthcare utilization and more [11]. These limitations argue for improved preclinical models to better determine SSRI impacts and reveal mechanistic insights. As we show here, preclinical work provides a powerful first step in understanding the impacts of antidepressants in pregnancy on brain health across the lifespan. Together, our results demonstrate that chronic, oral sertraline delivered via a naturalistic, non-invasive approach effectively modifies mood-related neurobiology and exerts lasting effects on maternal neurodegenerative processes, which may underlie dementia risk with advanced age. Interactions between SSRI exposure, pregnancy, and long-term brain health are complex and also require further study. We further find that pregnancy itself exerts long-term protective effects on neurodegenerative processes. If better understood, underlying mechanisms in pregnancy might be exploited to buffer against neurodegeneration. Our findings also highlight the need for additional, clinical studies to consider the long-term consequences of SSRI use and improve perinatal psychiatry guidelines.

Supplementary information is available at MP’s website.

## Data availibility

All data generated or analyzed during this study are included in this published article [and its supplementary information files].

## Supplementary information

Supplementary methods

Supplementary legends

Supplementary figure 1

Supplementary figure 2

Supplementary figure 3

Supplementary figure 4

Supplementary figure 5

Supplementary figure 6

Supplementary table 1

Supplementary table 2

## References

[1] Gaynes BN, Gavin N, Meltzer-Brody S, Lohr KN, Swinson T, Gartlehner G et al. Perinatal depression: prevalence, screening accuracy, and screening outcomes. Evid Rep Technol Assess (Summ). 2005:1–8. 10.1037/e439372005-001. 10.1037/e439372005-001 PMC4780910 15760246

[2] Lee AM, Lam SK, Sze Mun Lau SM, Chong CS, Chui HW, Fong DY. Prevalence, course, and risk factors for antenatal anxiety and depression. Obstet Gynecol. 2007;110:1102–12. 17978126 10.1097/01.AOG.0000287065.59491.70

[3] Davenport MH, Meyer S, Meah VL, Strynadka MC, Khurana R. Moms Are Not OK: COVID-19 and Maternal Mental Health. Front Glob Womens Health. 2020;1:1. 34816146 10.3389/fgwh.2020.00001 PMC8593957

[4] Trost SLBJ, Njie F, et al. Pregnancy-related deaths: data from maternal mortality review committees in 36 US States, 2017-2019. Centers for Disease Control and Prevention, US Department of Health and Human Services;2022.

[5] House W Blueprint for addressing the maternal health crisis. 2022.

[6] Brody D, Gu Q Antidepressant use among adults: United States, 2015–2018. *NCHS Data Brief, no 377 Hyattsville, MD: National Center for Health Statistics* 2020. 33054926

[7] Anderson KN, Lind JN, Simeone RM, Bobo WV, Mitchell AA, Riehle-Colarusso T, et al. Maternal use of specific antidepressant medications during early pregnancy and the risk of selected birth defects. JAMA Psychiatry. 2020;77:1246–55. 32777011 10.1001/jamapsychiatry.2020.2453 PMC7407327

[8] Cornet MC, Wu YW, Forquer H, Avalos LA, Sriram A, Scheffler AW, et al. Maternal treatment with selective serotonin reuptake inhibitors during pregnancy and delayed neonatal adaptation: a population-based cohort study. Arch Dis Child Fetal Neonatal Ed. 2024;109:294–300. 38071585 10.1136/archdischild-2023-326049 PMC11041605

[9] Vignato JA, Gumusoglu SB, Davis HA, Scroggins SM, Hamilton WS, Brandt DS et al. Selective serotonin reuptake inhibitor use in pregnancy and protective mechanisms in preeclampsia. Reprod Sci. 2023;30:701–12. 10.1007/s43032-022-01065-z PMC9944568 35984571

[10] Obermanns J, Flasbeck V, Steinmann S, Juckel G, Emons B. Investigation of the serotonergic activity and the serotonin content in serum and platelet, and the possible role of the serotonin transporter in patients with depression. Behav Sci (Basel). 2022;12:178. 35735388 10.3390/bs12060178 PMC9220674

[11] Gumusoglu SB, Schickling BM, Vignato JA, Santillan DA, Santillan MK. Selective serotonin reuptake inhibitors and preeclampsia: a quality assessment and meta-analysis. Pregnancy Hypertens. 2022;30:36–43. 35963154 10.1016/j.preghy.2022.08.001 PMC9712168

[12] Hou R, Ye G, Liu Y, Chen X, Pan M, Zhu F, et al. Effects of SSRIs on peripheral inflammatory cytokines in patients with Generalized Anxiety Disorder. Brain Behav Immun. 2019;81:105–10. 31163212 10.1016/j.bbi.2019.06.001

[13] Golyszny M, Obuchowicz E. Are neuropeptides relevant for the mechanism of action of SSRIs? Neuropeptides. 2019;75:1–17. 30824124 10.1016/j.npep.2019.02.002

[14] Więdłocha M, Marcinowicz P, Krupa R, Janoska-Jaździk M, Janus M, Dębowska W, et al. Effect of antidepressant treatment on peripheral inflammation markers - A meta-analysis. Prog Neuropsychopharmacol Biol Psychiatry. 2018;80:217–26. 28445690 10.1016/j.pnpbp.2017.04.026

[15] Lopez-Vilchez I, Diaz-Ricart M, Navarro V, Torramade S, Zamorano-Leon J, Lopez-Farre A, et al. Endothelial damage in major depression patients is modulated by SSRI treatment, as demonstrated by circulating biomarkers and an in vitro cell model. Transl Psychiatry. 2016;6:e886. 27598970 10.1038/tp.2016.156 PMC5048198

[16] Gur TL, Palkar AV, Rajasekera T, Allen J, Niraula A, Godbout J, et al. Prenatal stress disrupts social behavior, cortical neurobiology and commensal microbes in adult male offspring. Behav Brain Res. 2019;359:886–94. 29949734 10.1016/j.bbr.2018.06.025 PMC6542272

[17] Moncrieff J, Cooper RE, Stockmann T, Amendola S, Hengartner MP, Horowitz MA. The serotonin theory of depression: a systematic umbrella review of the evidence. Mol Psychiatry. 2022;28:3243–56. 35854107 10.1038/s41380-022-01661-0 PMC10618090

[18] Molenaar NM, Kamperman AM, Boyce P, Bergink V. Guidelines on treatment of perinatal depression with antidepressants: an international review. Aust N Z J Psychiatry. 2018;52:320–7. 29506399 10.1177/0004867418762057 PMC5871019

[19] Fischer Fumeaux CJ, Morisod Harari M, Weisskopf E, Eap CB, Epiney M, Vial Y, et al. Risk-benefit balance assessment of SSRI antidepressant use during pregnancy and lactation based on best available evidence - an update. Expert Opin Drug Saf. 2019;18:949–63. 31430189 10.1080/14740338.2019.1658740

[20] Dubovicky M, Belovicova K, Csatlosova K, Bogi E. Risks of using SSRI / SNRI antidepressants during pregnancy and lactation. Interdiscip Toxicol. 2017;10:30–4. 30123033 10.1515/intox-2017-0004 PMC6096863

[21] Hviid A, Melbye M, Pasternak B. Use of selective serotonin reuptake inhibitors during pregnancy and risk of autism. N Engl J Med. 2013;369:2406–15. 24350950 10.1056/NEJMoa1301449

[22] Grzeskowiak LE, Gilbert AL, Morrison JL. Long term impact of prenatal exposure to SSRIs on growth and body weight in childhood: evidence from animal and human studies. Reprod Toxicol. 2012;34:101–9. 22433946 10.1016/j.reprotox.2012.03.003

[23] van Geffen EC, Hermsen JH, Heerdink ER, Egberts AC, Verbeek-Heida PM, van Hulten R. The decision to continue or discontinue treatment: experiences and beliefs of users of selective serotonin-reuptake inhibitors in the initial months–a qualitative study. Res Social Adm Pharm. 2011;7:134–50. 21272543 10.1016/j.sapharm.2010.04.001

[24] Ratajczak P, Martynski J, Zieba JK, Swilo K, Kopciuch D, Paczkowska A, et al. Comparative efficacy of animal depression models and antidepressant treatment: a systematic review and meta-analysis. Pharmaceutics. 2024;16:1144. 39339181 10.3390/pharmaceutics16091144 PMC11435171

[25] Normann C, Frase S, Haug V, von Wolff G, Clark K, Munzer P, et al. Antidepressants rescue stress-induced disruption of synaptic plasticity via serotonin transporter-independent inhibition of L-type calcium channels. Biol Psychiatry. 2018;84:55–64. 29174591 10.1016/j.biopsych.2017.10.008

[26] Schoenfeld TJ, McCausland HC, Morris HD, Padmanaban V, Cameron HA. Stress and loss of adult neurogenesis differentially reduce hippocampal volume. Biol Psychiatry. 2017;82:914–23. 28629541 10.1016/j.biopsych.2017.05.013 PMC5683934

[27] McEwen BS, Gianaros PJ. Central role of the brain in stress and adaptation: links to socioeconomic status, health, and disease. Ann N Y Acad Sci. 2010;1186:190–222. 20201874 10.1111/j.1749-6632.2009.05331.x PMC2864527

[28] Jackson SJ, Andrews N, Ball D, Bellantuono I, Gray J, Hachoumi L, et al. Does age matter? The impact of rodent age on study outcomes. Lab Anim. 2017;51:160–9. 27307423 10.1177/0023677216653984 PMC5367550

[29] George B, Lumen A, Nguyen C, Wesley B, Wang J, Beitz J, et al. Application of physiologically based pharmacokinetic modeling for sertraline dosing recommendations in pregnancy. NPJ Syst Biol Appl. 2020;6:36. 33159093 10.1038/s41540-020-00157-3 PMC7648747

[30] Nair AB, Jacob S. A simple practice guide for dose conversion between animals and human. J Basic Clin Pharm. 2016;7:27–31. 27057123 10.4103/0976-0105.177703 PMC4804402

[31] DeVane CL, Liston HL, Markowitz JS. Clinical pharmacokinetics of sertraline. Clin Pharmacokinet. 2002;41:1247–66. 12452737 10.2165/00003088-200241150-00002

[32] Kino T. Stress, glucocorticoid hormones, and hippocampal neural progenitor cells: implications to mood disorders. Front Physiol. 2015;6:230. 26347657 10.3389/fphys.2015.00230 PMC4541029

[33] Baudat M, de Kort AR, van den Hove DLA, Joosten EA. Early-life exposure to selective serotonin reuptake inhibitors: long-term effects on pain and affective comorbidities. Eur J Neurosci. 2022;55:295–317. 34841582 10.1111/ejn.15544 PMC9299880

[34] Adriao A, Santana I, Ribeiro C, Cancela ML, Conceicao N, Grazina M. Identification of a novel mutation in MEF2C gene in an atypical patient with frontotemporal lobar degeneration. Neurol Sci. 2022;43:319–26. 33999292 10.1007/s10072-021-05269-0

[35] Aloni E, Oni-Biton E, Tsoory M, Moallem DH, Segal M. Synaptopodin deficiency ameliorates symptoms in the 3xTg mouse model of Alzheimer’s disease. J Neurosci. 2019;39:3983–92. 30872324 10.1523/JNEUROSCI.2920-18.2019 PMC6520517

[36] Barker SJ, Raju RM, Milman NEP, Wang J, Davila-Velderrain J, Gunter-Rahman F, et al. MEF2 is a key regulator of cognitive potential and confers resilience to neurodegeneration. Sci Transl Med. 2021;13:eabd7695. 34731014 10.1126/scitranslmed.abd7695 PMC9258338

[37] Ji C, Tang M, Zeidler C, Hohfeld J, Johnson GV. BAG3 and SYNPO (synaptopodin) facilitate phospho-MAPT/Tau degradation via autophagy in neuronal processes. Autophagy. 2019;15:1199–213. 30744518 10.1080/15548627.2019.1580096 PMC6613891

[38] Ren J, Zhang S, Wang X, Deng Y, Zhao Y, Xiao Y, et al. MEF2C ameliorates learning, memory, and molecular pathological changes in Alzheimer’s disease in vivo and in vitro. Acta Biochim Biophys Sin (Shanghai). 2022;54:77–90. 35130621 10.3724/abbs.2021012 PMC9909301

[39] Sunderaraman P, Cosentino S, Schupf N, Manly J, Gu Y, Barral S. MEF2C common genetic variation is associated with different aspects of cognition in non-hispanic white and caribbean hispanic non-demented older adults. Front Genet. 2021;12:642327. 34386032 10.3389/fgene.2021.642327 PMC8353395

[40] Zhang Z, Zhao Y. Progress on the roles of MEF2C in neuropsychiatric diseases. Mol Brain. 2022;15:8. 34991657 10.1186/s13041-021-00892-6 PMC8740500

[41] Wray NR, Ripke S, Mattheisen M, Trzaskowski M, Byrne EM, Abdellaoui A, et al. Genome-wide association analyses identify 44 risk variants and refine the genetic architecture of major depression. Nat Genet. 2018;50:668–81. 29700475 10.1038/s41588-018-0090-3 PMC5934326

[42] Howard DM, Adams MJ, Shirali M, Clarke TK, Marioni RE, Davies G, et al. Genome-wide association study of depression phenotypes in UK Biobank identifies variants in excitatory synaptic pathways. Nat Commun. 2018;9:1470. 29662059 10.1038/s41467-018-03819-3 PMC5902628

[43] Hyde CL, Nagle MW, Tian C, Chen X, Paciga SA, Wendland JR, et al. Identification of 15 genetic loci associated with risk of major depression in individuals of European descent. Nat Genet. 2016;48:1031–6. 27479909 10.1038/ng.3623 PMC5706769

[44] Gandal MJ, Haney JR, Parikshak NN, Leppa V, Ramaswami G, Hartl C, et al. Shared molecular neuropathology across major psychiatric disorders parallels polygenic overlap. Science. 2018;359:693–7. 29439242 10.1126/science.aad6469 PMC5898828

[45] Weber MA, Kerr G, Thangavel R, Conlon MM, Gumusoglu SB, Gupta K, et al. Alpha-Synuclein Pre-Formed Fibrils Injected into Prefrontal Cortex Primarily Spread to Cortical and Subcortical Structures. J Parkinsons Dis. 2024;14:81–94. 10.3233/JPD-230129 PMC10836574 38189765

[46] Bertolli A, Halhouli O, Liu-Martinez Y, Blaine B, Thangavel R, Zhang Q et al. Renovating the Barnes maze for mouse models of Dementia with STARR FIELD: A 4-day protocol that probes learning rate, retention and cognitive flexibility. *bioRxiv* [Preprint]. 2024 10.1101/2024.11.30.625516.

[47] Seno S, Tomura S, Miyazaki H, Sato S, Saitoh D. Effects of selective serotonin reuptake inhibitors on depression-like behavior in a laser-induced shock wave model. Front Neurol. 2021;12:602038. 33643190 10.3389/fneur.2021.602038 PMC7902879

[48] Mombereau C, Gur TL, Onksen J, Blendy JA. Differential effects of acute and repeated citalopram in mouse models of anxiety and depression. Int J Neuropsychopharmacol. 2010;13:321–34. 20003619 10.1017/S1461145709990630 PMC3646514

[49] Jacobsen JPR, Oh A, Bangle R, Roberts WL, Royer EL, Modesto N, et al. Slow-release delivery enhances the pharmacological properties of oral 5-hydroxytryptophan: mouse proof-of-concept. Neuropsychopharmacology. 2019;44:2082–90. 31035282 10.1038/s41386-019-0400-1 PMC6898594

[50] Heinonen E, Blennow M, Blomdahl-Wetterholm M, Hovstadius M, Nasiell J, Pohanka A, et al. Sertraline concentrations in pregnant women are steady and the drug transfer to their infants is low. Eur J Clin Pharmacol. 2021;77:1323–31. 33751155 10.1007/s00228-021-03122-z PMC8346399

[51] Sit DK, Perel JM, Helsel JC, Wisner KL. Changes in antidepressant metabolism and dosing across pregnancy and early postpartum. J Clin Psychiatry. 2008;69:652–8. 18426260 10.4088/jcp.v69n0419 PMC2408825

[52] Schoretsanitis G, Spigset O, Stingl JC, Deligiannidis KM, Paulzen M, Westin AA. The impact of pregnancy on the pharmacokinetics of antidepressants: a systematic critical review and meta-analysis. Expert Opin Drug Metab Toxicol. 2020;16:431–40. 32238008 10.1080/17425255.2020.1750598 PMC7323120

[53] Stika CS, Wisner KL, George AL Jr., Avram MJ, Zumpf K, Rasmussen-Torvik LJ, et al. Changes in sertraline plasma concentrations across pregnancy and postpartum. Clin Pharmacol Ther. 2022;112:1280–90. 36094046 10.1002/cpt.2746 PMC13103856

[54] Conti B, Maier R, Barr AM, Morale MC, Lu X, Sanna PP, et al. Region-specific transcriptional changes following the three antidepressant treatments electro convulsive therapy, sleep deprivation and fluoxetine. Mol Psychiatry. 2007;12:167–89. 17033635 10.1038/sj.mp.4001897

[55] Rayan NA, Kumar V, Aow J, Rastegar N, Lim MGL, O’Toole N, et al. Integrative multi-omics landscape of fluoxetine action across 27 brain regions reveals global increase in energy metabolism and region-specific chromatin remodelling. Mol Psychiatry. 2022;27:4510–25. 36056172 10.1038/s41380-022-01725-1 PMC9734063

[56] Chaves T, Fazekas CL, Horvath K, Correia P, Szabo A, Torok B, et al. Stress adaptation and the brainstem with focus on corticotropin-releasing hormone. Int J Mol Sci. 2021;22:9090. 34445795 10.3390/ijms22169090 PMC8396605

[57] Qiu J, Yao S, Hindmarch C, Antunes V, Paton J, Murphy D. Transcription factor expression in the hypothalamo-neurohypophyseal system of the dehydrated rat: upregulation of gonadotrophin inducible transcription factor 1 mRNA is mediated by cAMP-dependent protein kinase A. J Neurosci. 2007;27:2196–203. 17329416 10.1523/JNEUROSCI.5420-06.2007 PMC6673476

[58] Shi Z, Pelletier NE, Wong J, Li B, Sdrulla AD, Madden CJ, et al. Leptin increases sympathetic nerve activity via induction of its own receptor in the paraventricular nucleus. Elife. 2020;9:e55357. 32538782 10.7554/eLife.55357 PMC7316512

[59] Kageyama K, Suda T. Regulatory mechanisms underlying corticotropin-releasing factor gene expression in the hypothalamus. Endocr J. 2009;56:335–44. 19352056 10.1507/endocrj.k09e-075

[60] Herman JP, Tasker JG. Paraventricular hypothalamic mechanisms of chronic stress adaptation. Front Endocrinol (Lausanne). 2016;7:137. 27843437 10.3389/fendo.2016.00137 PMC5086584

[61] Yuan PQ, Yang H. Neuronal activation of brain vagal-regulatory pathways and upper gut enteric plexuses by insulin hypoglycemia. Am J Physiol Endocrinol Metab. 2002;283:E436–48. 12169436 10.1152/ajpendo.00538.2001 PMC8091863

[62] Jeong JK, Dow SA, Young CN. Sensory circumventricular organs, neuroendocrine control, and metabolic regulation. Metabolites. 2021;11:494. 34436435 10.3390/metabo11080494 PMC8402088

[63] Croese T, Ramos, JM, Castellani, G, Ferrera, S, Tzipora, FZ, Judith, AP, and Schwartz, M Pregnancy exacerbates early-onset familial Alzheimer’s disease. Neurology 2021;96:4075.

[64] Cummings M, Arumanayagam ACS, Zhao P, Kannanganat S, Stuve O, Karandikar NJ, et al. Presenilin1 regulates Th1 and Th17 effector responses but is not required for experimental autoimmune encephalomyelitis. PLoS One. 2018;13:e0200752. 30089166 10.1371/journal.pone.0200752 PMC6082653

[65] Li XY, Wang F, Chen GH, Li XW, Yang QG, Cao L, et al. Inflammatory insult during pregnancy accelerates age-related behavioral and neurobiochemical changes in CD-1 mice. Age (Dordr). 2016;38:59. 27194408 10.1007/s11357-016-9920-3 PMC5005951

[66] Pittenger C, Duman RS. Stress, depression, and neuroplasticity: a convergence of mechanisms. Neuropsychopharmacology. 2008;33:88–109. 17851537 10.1038/sj.npp.1301574

[67] Johansen A, Armand S, Plaven-Sigray P, Nasser A, Ozenne B, Petersen IN, et al. Effects of escitalopram on synaptic density in the healthy human brain: a randomized controlled trial. Mol Psychiatry. 2023;28:4272–9. 37814129 10.1038/s41380-023-02285-8 PMC10827655

[68] Duan W, Peng Q, Masuda N, Ford E, Tryggestad E, Ladenheim B, et al. Sertraline slows disease progression and increases neurogenesis in N171-82Q mouse model of Huntington’s disease. Neurobiol Dis. 2008;30:312–22. 18403212 10.1016/j.nbd.2008.01.015 PMC3683653

[69] Seeburg DP, Feliu-Mojer M, Gaiottino J, Pak DT, Sheng M. Critical role of CDK5 and Polo-like kinase 2 in homeostatic synaptic plasticity during elevated activity. Neuron. 2008;58:571–83. 18498738 10.1016/j.neuron.2008.03.021 PMC2488274

[70] Spilker C, Grochowska KM, Kreutz MR. What do we learn from the murine Jacob/Nsmf gene knockout for human disease? Rare Dis. 2016;4:e1241361. 27803842 10.1080/21675511.2016.1241361 PMC5070631

[71] Wegener S, Buschler A, Stempel AV, Kang SJ, Lim CS, Kaang BK, et al. Defective synapse maturation and enhanced synaptic plasticity in Shank2 Deltaex7(-/-) Mice. eNeuro. 2018;5:ENEURO.0398-17.2018. 30023428 10.1523/ENEURO.0398-17.2018 PMC6049608

[72] Monteiro P, Feng G. SHANK proteins: roles at the synapse and in autism spectrum disorder. Nat Rev Neurosci. 2017;18:147–57. 28179641 10.1038/nrn.2016.183

[73] Barbosa AC, Kim MS, Ertunc M, Adachi M, Nelson ED, McAnally J, et al. MEF2C, a transcription factor that facilitates learning and memory by negative regulation of synapse numbers and function. Proc Natl Acad Sci USA. 2008;105:9391–6. 18599438 10.1073/pnas.0802679105 PMC2453723

[74] Farsi Z, Nicolella A, Simmons SK, Aryal S, Shepard N, Brenner K, et al. Brain-region-specific changes in neurons and glia and dysregulation of dopamine signaling in Grin2a mutant mice. Neuron. 2023;111:3378–96.e3379. 37657442 10.1016/j.neuron.2023.08.004

[75] Ammari R, Monaca F, Cao M, Nassar E, Wai P, Del Grosso NA, et al. Hormone-mediated neural remodeling orchestrates parenting onset during pregnancy. Science. 2023;382:76–81. 37797007 10.1126/science.adi0576 PMC7615220

[76] Chan RW, Ho LC, Zhou IY, Gao PP, Chan KC, Wu EX. Structural and functional brain remodeling during pregnancy with diffusion tensor MRI and resting-state functional MRI. PLoS One. 2015;10:e0144328. 26658306 10.1371/journal.pone.0144328 PMC4675543

[77] Hillerer KM, Jacobs VR, Fischer T, Aigner L. The maternal brain: an organ with peripartal plasticity. Neural Plast. 2014;2014:574159. 24883213 10.1155/2014/574159 PMC4026981

[78] Zhou R, Liu HM, Zou LW, Wei HX, Huang YN, Zhong Q, et al. Associations of parity with change in global cognition and incident cognitive impairment in older women. Front Aging Neurosci. 2022;14:864128. 35601623 10.3389/fnagi.2022.864128 PMC9114765

[79] Bartels C, Wagner M, Wolfsgruber S, Ehrenreich H, Schneider A. Alzheimer’s disease neuroimaging I. Impact of SSRI therapy on risk of conversion from mild cognitive impairment to Alzheimer’s dementia in individuals with previous depression. Am J Psychiatry. 2018;175:232–41. 29179578 10.1176/appi.ajp.2017.17040404

[80] Rajkumar R. Resolving a paradox: antidepressants, neuroinflammation, and neurodegeneration. Explor Neuroprot Ther. 2024;4:11–37.

[81] Reed MB, Vanicek T, Seiger R, Klobl M, Spurny B, Handschuh P, et al. Neuroplastic effects of a selective serotonin reuptake inhibitor in relearning and retrieval. Neuroimage. 2021;236:118039. 33852940 10.1016/j.neuroimage.2021.118039 PMC7610799

[82] Sun DS, Gao LF, Jin L, Wu H, Wang Q, Zhou Y, et al. Fluoxetine administration during adolescence attenuates cognitive and synaptic deficits in adult 3xTgAD mice. Neuropharmacology. 2017;126:200–12. 28911966 10.1016/j.neuropharm.2017.08.037

[83] Castagne V, Moser P, Roux S, Porsolt RD Rodent models of depression: forced swim and tail suspension behavioral despair tests in rats and mice. Curr Protoc Neurosci 2011; Ch. 8: Unit 8.10A. 10.1002/0471142301.ns0810as55 21462162

[84] Sensini F, Inta D, Palme R, Brandwein C, Pfeiffer N, Riva MA, et al. The impact of handling technique and handling frequency on laboratory mouse welfare is sex-specific. Sci Rep. 2020;10:17281. 33057118 10.1038/s41598-020-74279-3 PMC7560820

[85] Clarkson JM, Dwyer DM, Flecknell PA, Leach MC, Rowe C. Handling method alters the hedonic value of reward in laboratory mice. Sci Rep. 2018;8:2448. 29402923 10.1038/s41598-018-20716-3 PMC5799408

[86] Ueno H, Takahashi Y, Suemitsu S, Murakami S, Kitamura N, Wani K, et al. Effects of repetitive gentle handling of male C57BL/6NCrl mice on comparative behavioural test results. Sci Rep. 2020;10:3509. 32103098 10.1038/s41598-020-60530-4 PMC7044437

[87] Chiba S, Numakawa T, Ninomiya M, Richards MC, Wakabayashi C, Kunugi H. Chronic restraint stress causes anxiety- and depression-like behaviors, downregulates glucocorticoid receptor expression, and attenuates glutamate release induced by brain-derived neurotrophic factor in the prefrontal cortex. Prog Neuropsychopharmacol Biol Psychiatry. 2012;39:112–9. 22664354 10.1016/j.pnpbp.2012.05.018

[88] Gumusoglu SB, Maurer SV, Stevens HE. Dataset describing maternal prenatal restraint stress effects on immune factors in mice. Data Brief. 2022;43:108348. 35707242 10.1016/j.dib.2022.108348 PMC9189994

[89] Gumusoglu S, Scroggins S, Vignato J, Santillan D, Santillan M. The serotonin-immune axis in preeclampsia. Curr Hypertens Rep. 2021;23:37. 34351543 10.1007/s11906-021-01155-4 PMC8435353

[90] Gumusoglu S, Meincke CR, Kiel M, Betz A, Nuckols V, DuBose L, et al. Low indoleamine 2, 3 dioxygenase (IDO) activity is associated with psycho-obstetric risk. Pregnancy Hypertens. 2024;35:12–8. 38064980 10.1016/j.preghy.2023.11.008 PMC11003651

[91] Mertens M Protect Pregnant Women ‘Through Research,’ Not ‘From Research,’ OB-GYNs Urge. npr.: Shots Health News From NPR, 2021.

[92] Gumusoglu SB, Chilukuri ASS, Hing BWQ, Scroggins SM, Kundu S, Sandgren JA, et al. Altered offspring neurodevelopment in an arginine vasopressin preeclampsia model. Transl Psychiatry. 2021;11:79. 33510137 10.1038/s41398-021-01205-0 PMC7844013

[93] Zoega H, Kieler H, Norgaard M, Furu K, Valdimarsdottir U, Brandt L, et al. Use of SSRI and SNRI Antidepressants during Pregnancy: A Population-Based Study from Denmark, Iceland, Norway and Sweden. PLoS One. 2015;10:e0144474. 26657647 10.1371/journal.pone.0144474 PMC4685993

[94] Wikman A, Skalkidou A, Wikstrom AK, Lampa E, Kramer MS, Yong EL, et al. Factors associated with re-initiation of antidepressant treatment following discontinuation during pregnancy: a register-based cohort study. Arch Womens Ment Health. 2020;23:709–17. 32632522 10.1007/s00737-020-01050-y PMC7497307

