# Supplementary material for: Peripartum sertraline impacts maternal neurobehavioral and neurodegenerative mechanisms in pregnant and postpartum mice
Source: Mol Psychiatry. 2025 Jul 18;30(11):5108–20. doi: 10.1038/s41380-025-03094-x (PMC12532605; doi:10.1038/s41380-025-03094-x)
Supplement: Supplementary file 1 — Supplementary methods [file 41380_2025_3094_MOESM1_ESM.docx]

**Supplement**

**EXTENDED METHODS AND MATERIALS:**

**Mice:** C56Bl6/J mice (Jackson Labs, Bar Harbor, ME, USA) approximately 6-8 weeks of age were given sertraline or water via in-cage water bottles. Animals were individually housed during pregnancy and lactation but otherwise were group-housed 3-4 per cage. Animals were considered pregnant upon detection of a vaginal plug [gestational day (GD) 0] and 15 days following the start of sertraline administration was experimental day 0 (ED 0) in non-pregnant animals. To allow for comparison with prior and ongoing studies in similar pregnancy models, all females were implanted with subcutaneous osmotic minipumps (Alzet #1004) filled with sterile saline then were successfully bred to syngenic, age-matched males within a 5-day breeding period, or were unbred (non-pregnant controls) for the same duration. Only females were tested. Housing occurred under regular conditions: lights on/off at 0900/2100 DST with an average ambient temperature of 22 °C and food and water *ad libitum* unless described differently below. All procedures involving animals were approved by University of Iowa Institutional Animal Care and Use Committee. Sample sizes are detailed in each figure and in the relevant results sections. Unbred, virgin females of an equivalent age and background were used as non-pregnant controls (Fig. 1A for timeline of experiments). Animals were randomly selected for all experiments unless otherwise noted.

**Sertraline administration:** Sertraline (167 mg/L oral suspension; NorthstarRx LLC Pharmaceuticals, Memphis, TN, USA) was administered *ad libitum* via in-cage water bottles starting two weeks prior to breeding then for the duration of pregnancy and lactation until weaning, or for an equivalent duration (approximately 8 weeks) in non-pregnant animals. Sertraline animals did not have access to house water during drug treatment. Control animals were given untreated water via in-cage water bottles. Bedding, light/dark cycle, and other housing particulars are described previously ^1, 2^.

**Liquid chromatography–mass spectrometry/mass spectrometry (LC-MS/MS)*:*** Plasma samples (50 µl) were placed in microcentrifuge tubes along with 50 µL of 0.1 M NaOH solution, 5 µL of 100 ng/ml Setraline-d3 (internal standard) stock solution, and 500 µL Ethyl Acetate for extraction. The mixture was vortexed for 15 minutes then centrifuged 4000 rpm for 10 minutes at 4°C. The supernatant was carefully removed to a glass tube. Another 500 µL Ethyl Acetate was added to the original microcentrifuge tube for a second extraction. The samples were again vortexed and centrifuged. The supernatants were combined in the glass tube. Ethyl Acetate was evaporated under a gentle stream of high purity nitrogen at 50°C until almost dry. The samples were reconstituted in 50 µl of 1:1(v/v) Water/Acetonitrile with 0.1% Formic Acid and centrifuged at 4000 rpm for 10 minutes. Forty microliters were moved to an autosampler vial for LC-MS/MS analysis.

LC-MS/MS data were collected on a Water Xevo TQ-S cronos with an Acquity H-Class UPLC system. The LC column (Waters Acquity BEH C18, 2.1 mm x 100 mm, 1.8 µm) was held at 40°C throughout the run. Flow rate was 0.3 ml/min. Injection volume was 5 µl. For the mobile phase, solvent A was Water with 0.1% Formic Acid and solvent B was Acetonitrile with 0.1% Formic Acid. The LC gradient started at 50% Solvent B and increased over 5 minutes to 100%, where it was held for 4 minutes prior to re-equilibration. Data were collected using positive electrospray. The (M+H)+ ion to product ion (selected reaction monitoring or SRM) transitions used were 305.98 > 275.02 and 309.0 > 275.12 for Sertraline and Setraline-d3, respectively.

**General animal and pregnancy health assessments:** To confirm no preference or lack of preference for sertraline-treated water, animals were individually housed and monitored for water and sertraline-treated water consumption by daily water bottle weight checks for the first two days of sertraline administration. Animals were weighed at study outset, GD 0, 12, and 18 [or at experimental day (ED) equivalent if unbred]. As previously ^3, 4^, on GD 16 or ED 16, urine was collected from females (minimum 100 µL) and tested for protein concentration to confirm normal kidney function. Total protein was assayed according to manufacturer's instructions via the commercially available bicinchoninic acid (BCA) colorimetric total protein assay kit (Thermo Scientific, Rockford, IL, USA). Pups were counted on the day of birth (P0) to determine litter size.

Blood pressure was measured as previously ^3, 4^ via tail cuff plethysmography (CODA, Kent Scientific, Torrington, CT, USA) in a separate cohort from those used for molecular and behavioral studies. Before testing, mice were acclimated to testing for 2 weeks. Mice were restrained in cylindrical, plexiglass restraints during plethysmography. Pregnant mice were tested at GD16-18 and values averaged for report. Non-pregnant mice were tested at the equivalent experimental days and values were averaged over 2 days of testing (GD 16-18 or ED 16-18).

**Serotonin ELISA:** Peripheral, platelet-free serotonin levels were assessed via commercially available enzyme-linked immunosorbent assay (ELISA) kits (Abcam, ab133053), as per manufacturer instructions. Samples were assessed at GD 0, 5, 12, and 18. To collect platelet-free plasma, blood was collected into heparinized tubes, centrifuged (2000g for 10 min), plasma transferred and centrifuged (4000g for 20 min), then snap frozen to -20°C until assayed by ELISA.

**Behavioral testing:** All experiments were designed with animal welfare at the fore. Animals were monitored throughout to confirm good health and well-being. All experiments were conducted during the animals' light cycle. Materials were cleaned (70% ethanol) between trials. Each behavioral assay was conducted on a separate day, with tests run and detailed below in the following order: forced swim test (FST), tail suspension test (TST), elevated plus maze (EPM), open field test (OFT), chocolate preference, Barnes maze, and radial arm maze. Experimenters were blinded to animal condition throughout all behavioral testing.

**Forced swim test:** As previously^5^, mice were individually placed in a clear acrylic cylinder (outer diameter: 23 cm; inner diameter: 21.5 cm, height: 34 cm) which was filled halfway with 20-25°C water and video recorded for 6 minutes. Videos were recorded such that the animal’s head, all four limbs, and tail were readily visualizable. Trials were coded for total swim time by two blinded experimenters and the two times averaged. Coordinated movement of two or more limbs was considered mobile behavior.

**Tail suspension test:** Following standard protocols and as previously^5^, mice were suspended by the tail approximately 20 inches above a table for six minutes. Animals were recorded such that all limbs and the full extent of their body was visible. Trials were coded for struggle time by two blinded experimenters and the two averaged together. Struggle time was defined as any movement of both limbs or body.

**Elevated plus maze:** As previously ^1, 6, 7^, mice were placed in the center of an elevated plus maze (~250 lux in open arms) oriented towards a closed arm. Mice were allowed to freely explore the maze for five minutes. All movement was recorded via overhead camera and tracked by EthoVision software (Noldus, Leesburg, VA, USA) for number of visits to the open arm.

**Open field test:** As previously ^1, 2, 5-7^ , mice were placed in the center of an open field arena (16 x 16.5 x 12 in, black acrylic, 100 lux in center) and allowed to freely explore for 30 minutes. EthoVision software was used to measure time spent in the center versus the periphery of the field (each defined to include 50% of surface area) ^7^.

**Chocolate preference:** In-cage food hoppers were modified to allow separation into two, equivalently-sized baskets. Each animal was given equivalent amounts of standard chow or milk chocolate nuggets (Hershey’s Kisses) and consumption of each was measured by weight across 12-hour epochs for 48 consecutive hours. No signs of toxicity or adverse effects of chocolate consumption were observed. The chocolate treats used have considerably lower levels of theobromine than might be considered toxic. Theobromine is markedly less toxic in rats and mice (LD 50: 1,000 mg/kg) than in other animals such as dogs (LD 50: 100-200 mg/kg).^8, 9^

**Barnes maze:** As previously ^10^, animals were tested across three days on a custom-made white plexiglass circular platform (122 cm diameter) with 40 portal holes around its periphery, 10 of which were escape portals which could be opened to allow animal access to a plexiglass escape holding shuttle below. To minimize animal stress and encourage a preference for the shuttle, upon entering the shuttle it was gently removed and placed within the animal’s home cage to allow for home cage return and a 3-minute break interval. The platform is fixed to a white plastic drum (55 gallon, 33 inches tall, 26.5 inches wide) on a turntable to allow for rotation. Caps were used to partially occlude portals and prevent animal visualization from the center of the table. Animal behavior was recorded throughout via overhead camera. The experimenter remained hidden throughout testing.

On day 1 of testing, the animal was placed in the center of the apparatus and allowed to explore the maze for a habituation trial. During this time, one escape portal allowed access to the holding shuttle, which contained home cage bedding to promote animal preference. This habituation trial was followed by 5 test trials with the same target portal location. All trials concluded upon animal arrival to the shuttle or at 10 minutes, whichever came first. All equipment were cleaned and dried thoroughly between trials and the table is rotated but target portal is held in the same relative position.

On day 2 of testing, first a probe trial is administered during which all portals are covered for 90 seconds. Following this probe were 6 typical test trials with the same target portal location as on day 1.

Day 3 of testing began with a probe trial and 6 typical test trials, as on day 2. All protocols were consistent with those described above.

**Barnes maze analyses:** All videos were analyzed by Any-Maze software (Stoelting Co., Wood Dale, IL USA). Distance to target was calculated as the total distance traveled until the animal’s nose reached the target portal. Test trials were averaged together for each day of testing.

**Radial arm maze:** One week after conclusion of Day 3 of the Barnes maze, a radial arm maze test was completed on the same apparatus utilized for the Barnes maze. A total of ten trials were conducted. During the first trial, the animal was allowed to explore the table without barriers for 90 seconds. Clear plastic barriers (0.250 inches thick) were then placed between targets and a target escape portal randomly selected for trials 2-5. It was ensured that this new target portal was at least 2 portals removed from the Barnes maze escape portal used previously with rotation and cleaning between trials to eliminate a scent path, as described above. For trials 6-9, a new target portal was designated and rotation and cleaning between trials continued.

Working memory errors were analyzed via automated review and cross-validated by manual scoring of a subset. Assessments calculated mouse visits to the center and outer zones of the table, and to the escape portal. Travel to the center, outer, then escape portal was counted as a visit. Re-checking of a previously checked portal (or serial checking) were counted as an error. Errors were analyzed for trial 10.

**Code availability:** Working memory errors were analyzed via automated review of videos. Custom code for this step is available upon request without restriction.

**Tissue collection:** Animals were sacrificed and tissues collected approximately 3 weeks following Barnes Maze testing. Mice were euthanized via CO_2_ overdose and rapid decapitation. Brains were dissected and hemi sectioned at the midline; one hemisphere was placed in 4% PFA and then cryopreserved in 20% sucrose for 24 hours before embedded in Optimal Cutting Temperature (OCT) compound and frozen to -80°C for coronal cryosectioning at 50 [μm](https://www.bing.com/ck/a?!&&p=720ee5699532e3b2JmltdHM9MTcyNjAxMjgwMCZpZ3VpZD0wN2FkY2JiYi1iNGJlLTZhMGQtMjNkMC1kZmVlYjUxNjZiZGEmaW5zaWQ9NTU0MA&ptn=3&ver=2&hsh=3&fclid=07adcbbb-b4be-6a0d-23d0-dfeeb5166bda&u=a1L3NlYXJjaD9xPU1pY3JvbWV0cmUmRk9STT1TTkFQU1QmZmlsdGVycz1zaWQ6IjA2YTM0ZGM1LWQ3ZTctMTc3NC0yNzY2LTBjODk0ODQ5NmUwMCI&ntb=1) into 1x PBS and 0.05% sodium azide. These were later slide mounted (Superfrost Plus, Fisher) for immunohistochemistry studies.

Complementary hemispheres from each animal were sectioned at 100 [μm](https://www.bing.com/ck/a?!&&p=720ee5699532e3b2JmltdHM9MTcyNjAxMjgwMCZpZ3VpZD0wN2FkY2JiYi1iNGJlLTZhMGQtMjNkMC1kZmVlYjUxNjZiZGEmaW5zaWQ9NTU0MA&ptn=3&ver=2&hsh=3&fclid=07adcbbb-b4be-6a0d-23d0-dfeeb5166bda&u=a1L3NlYXJjaD9xPU1pY3JvbWV0cmUmRk9STT1TTkFQU1QmZmlsdGVycz1zaWQ6IjA2YTM0ZGM1LWQ3ZTctMTc3NC0yNzY2LTBjODk0ODQ5NmUwMCI&ntb=1) using a plexiglass mold and stainless-steel razor blades. Sections were placed on glass slides and frozen at -80°C for later tissue punch collection and molecular studies. Punches were taken of pre-frontal cortex, hippocampus, and hypothalamic paraventricular nucleus with reference to the Allen Brain Atlas coronal mouse atlas^11^.

**RNA sequencing and differential gene expression analyses:** RNA sequencing was performed by using the Illumina HiSeq platform with a 2x150 bp paired-end library and PolyA selection (Genewiz, Azenta). Raw sequence reads were processed using Trimmomatic v0.36 to trim potential adapter sequences and low-quality bases. The resulting high-quality reads were aligned to the *Mus musculus* GRCm38 reference genome (ENSEMBL) using STAR aligner v2.5.2b. RNA sequencing mapping success rate approximated to >95%. Total number of genes considered in background gene set were 17820 for cortex, and 18448 for PVN. Gene-level quantification was carried out with featureCounts from the Subread package v1.5.2. Differential gene expression analysis was conducted using DESeq2, applying the Wald test to obtain p-values and log2 fold changes. Genes were considered differentially expressed if they had an adjusted p-value < 0.05 and an absolute log2 fold change > 1.

**Gene ontology and over-representation analyses:** Differentially expressed genes (DEGs) from maternal frontal cortex and paraventricular nucleus were tested for enrichment of gene ontology pathway-based sets curated from Reactome, Wikipathways, and KEGG via the Consensus Pathway Database (CPDB; Max Planck Institute). List of pathways provided as a supplement. Significant overlap between DEGs and Alzheimer’s Disease (AD) target genes was also tested. AD genes were identified by the National Institute on Aging’s Accelerating Medicines Partnership in Alzheimer’s Disease (AMP-AD) consortium and overlap tested by chi-square. Target risk scores (sum of multiomic and genetic risk scores, range 0-5), multiomic risk scores (summary score of transcriptomic and proteomic studies supporting association of target gene with late-onset AD, range 0-2), and genetic risk scores (summary score of genetic evidence supporting target gene’s association with late-onset AD drawn from genetic and phenotypic evidence from human and model organism studies, range 0-3) are given. FC and P values are provided for comparison and are derived from inferior frontal gyrus expression in AD. AD scores and values are available from agora.adknowledgeportal.org (downloaded: 8/16/24). Sequencing data used for these analyses are generated from over 2100 samples from post-mortem brains of more than 1100 individuals across three human cohort studies.

**RNA isolation and QPCR:** RNA samples were prepared and isolated from frozen tissue punch samples using a TRIzol (Invitrogen) protocol ^12^. RNA yields and quality were assessed using a NanoDrop (Thermo Fisher Scientific). cDNA was synthesized using the AMV Reverse Transcriptase cDNA Synthesis Kit (MO277S) ^12^. qPCR was performed with Power SYBR Green Master Mix (Thermo Fisher Scientific, Warrington, UK) and primers, as previously (Supplementary Table 1). Samples were run (ViiA 7 Real-Time PCR System) and gene expression was assessed in triplicate, calculated from average Ct values normalized to GAPDH (formula: 2^(−Ct)^), as previously ^1, 7, 13^.

**Immunohistochemistry:** Stain buffer for both primary and secondary steps was prepared as follows: 2% Triton 1 X-100, 1% tween, 20% Horse Serum in 1xPBS. Buffer was applied to fixed sections for 30 minutes at room temperature on dual action shaker. Sections were immunostained with two primary antibodies: VGlut1 (1:1000 Millipore AB5905) and PSD-95 (1:200, Millipore AB9708). Antibodies were left on sections overnight at 4°C, followed by three, 5-minute washes, and then incubation with respective secondary antibodies (1:500 goat-anti-guinea pig 488 green; Alexa Fluor A11073 and 1:500 goat-anti-rabbit 594 red; Alexa Fluor A11012) for 90 minutes at room temperature. After washing, slides were cover-slipped with DAPI mounting medium (4′,6-diamidino-2-phenylindole, H200010, Vector Laboratories, #H-1200). Photomicrographs at the level of the hippocampal CA3 region (reference to the Allen Brain Coronal Mouse Atlas) at 20x were obtained (Zeiss AxioImager 2) to quantify synaptic density.

**Stereology:** Photomicrographs of cortex, hippocampus, and corpus callosum were obtained at 10x (Olympus Upright BX61VS Compound Fluorescence Microscope). Contours were traced on Image J ^14^ (reference to the Allen Brain Coronal Mouse Atlas). Colocalized Vglut1 and PSD95 puncta were assessed using ImageJ’s “Fiji: Puncta Analyzer” plugin ^15^.

**Statistical Analyses:** Histologic, behavioral, and gene expression data were analyzed and graphed using GraphPad Prism 10.2 (GraphPad Software, San Diego, CA). Data were graphically represented as mean +/- standard error of the mean (SEM) for each group. Results were considered significant when *p*<0.05. Benjamini, Krieger and Yekutieli method of test correction was applied to all post-hoc t tests to derive an adjusted q value. Two-way ANOVAs and Bonferroni’s multiple comparisons tests were also used and corrected p values are reported for multiple comparison tests, as appropriate. For two-sided, two-sample t-tests, the assumption of homogeneity of variances was met. Statistical outliers were calculated as greater than two standard deviations from the mean and were excluded. P values <0.05 were significant and <0.1 were considered trends and are provided on figures, as appropriate. Sample sizes were selected based on our prior work in similar models ^1, 7^ or with similar outcomes.

**References:**

1. Gumusoglu SB, Hing BWQ, Chilukuri ASS, Dewitt JJ, Scroggins SM, Stevens HE. Chronic maternal interleukin-17 and autism-related cortical gene expression, neurobiology, and behavior. *Neuropsychopharmacology* 2020; **45**(6)**:** 1008-1017.

2. Santillan MK, Santillan DA, Scroggins SM, Min JY, Sandgren JA, Pearson NA *et al.* Vasopressin in preeclampsia: a novel very early human pregnancy biomarker and clinically relevant mouse model. *Hypertension* 2014; **64**(4)**:** 852-859.

3. Gumusoglu SB, Kiel MD, Gugel A, Schickling BM, Weaver KR, Lauffer MC *et al.* Anti-angiogenic mechanisms and serotonergic dysfunction in the Rgs2 knockout model for the study of psycho-obstetric risk. *Neuropsychopharmacology* 2023.

4. Sandgren JA, Deng G, Linggonegoro DW, Scroggins SM, Perschbacher KJ, Nair AR *et al.* Arginine vasopressin infusion is sufficient to model clinical features of preeclampsia in mice. *JCI Insight* 2018; **3**(19).

5. Lauffer M, Wen H, Myers B, Plumb A, Parker K, Williams A. Deletion of the voltage-gated calcium channel, Ca(V) 1.3, causes deficits in motor performance and associative learning. *Genes Brain Behav* 2022; **21**(2)**:** e12791.

6. Gumusoglu SB, Fine RS, Murray SJ, Bittle JL, Stevens HE. The role of IL-6 in neurodevelopment after prenatal stress. *Brain Behav Immun* 2017; **65:** 274-283.

7. Gumusoglu SB, Kiel MD, Gugel A, Schickling BM, Weaver KR, Lauffer MC *et al.* Anti-angiogenic mechanisms and serotonergic dysfunction in the Rgs2 knockout model for the study of psycho-obstetric risk. *Neuropsychopharmacology* 2024; **49**(5)**:** 864-875.

8. Tarka SM, Jr., Morrissey RB, Apgar JL, Hostetler KA, Shively CA. Chronic toxicity/carcinogenicity studies of cocoa powder in rats. *Food Chem Toxicol* 1991; **29**(1)**:** 7-19.

9. Nomura S, Monobe M. Comparing Effects of Caffeine and Theobromine on Adrenal Hypertrophy and Social Behavior in Psychosocial Stressed Mice. *J Nutr Sci Vitaminol (Tokyo)* 2023; **69**(2)**:** 145-149.

10. Weber MA, Kerr G, Thangavel R, Conlon MM, Gumusoglu SB, Gupta K *et al.* Alpha-Synuclein Pre-Formed Fibrils Injected into Prefrontal Cortex Primarily Spread to Cortical and Subcortical Structures. *J Parkinsons Dis* 2024; **14**(1)**:** 81-94.

11. Allen Reference Atlas – Mouse Brain [brain atlas]. Available from atlas.brain-map.org.

12. Elser BA, Kayali K, Dhakal R, O'Hare B, Wang K, Lehmler HJ *et al.* Combined Maternal Exposure to Cypermethrin and Stress Affect Embryonic Brain and Placental Outcomes in Mice. *Toxicol Sci* 2020; **175**(2)**:** 182-196.

13. Gumusoglu SB, Chilukuri ASS, Hing BWQ, Scroggins SM, Kundu S, Sandgren JA *et al.* Altered offspring neurodevelopment in an arginine vasopressin preeclampsia model. *Transl Psychiatry* 2021; **11**(1)**:** 79.

14. Schneider CA, Rasband WS, Eliceiri KW. NIH Image to ImageJ: 25 years of image analysis. *Nat Methods* 2012; **9**(7)**:** 671-675.

15. Schindelin J, Arganda-Carreras I, Frise E, Kaynig V, Longair M, Pietzsch T *et al.* Fiji: an open-source platform for biological-image analysis. *Nat Methods* 2012; **9**(7)**:** 676-682.
