# Supplementary material for: Peripartum sertraline impacts maternal neurobehavioral and neurodegenerative mechanisms in pregnant and postpartum mice
Source: Mol Psychiatry. 2025 Jul 18;30(11):5108–20. doi: 10.1038/s41380-025-03094-x (PMC12532605; doi:10.1038/s41380-025-03094-x)
Supplement: Supplementary file 2 — Supplementary legends [file 41380_2025_3094_MOESM2_ESM.docx]

**SUPPLEMENTARY FIGURE LEGENDS:**

**Supplementary Figure 1:** A) Exemplar LC-MS/MS chromatograms for Sertraline-d3 and B) for Sertraline in a plasma sample. The chromatograms show the integrated peaks from MRM transitions 309→275 and 305.9→275, respectively, that were used for quantification. Exemplar chromatographic peaks from maternal plasma sample from sertraline-treated mouse validates detection of sertraline relative to a d3-labeled sertraline internal control. C, D) Animals did not drink differential amounts of sertraline-treated versus untreated water over either of two days of assessment. E) Urine protein is not changed by sertraline administration in late pregnancy. F) Number of pups born is also not changed by sertraline administration. G) Systolic blood pressure is not changed by pregnancy (GD16-18 averaged) nor by sertraline administration. H) No significant difference in total distance traveled on the OFT apparatus regardless of SSRI or pregnancy status. I, J) Hedonic behavior was measured during pregnancy from GD12 to GD18. Total consumption of chow and a high-sugar treat (chocolate kisses) were measured over a period of 24 hours. I) Non-pregnant animals consumed significantly more chow (two-way ANOVA main effect of pregnancy: F[1, 14] = 17.14; posthoc **p*=0.001) and J) chocolate (two-way ANOVA main effect of pregnancy: F[1, 14] = 138.0; posthoc **p*=<0.0001), regardless of sertraline status.

**Supplementary Figure 2:** Interaction analyses of PVN and cortical DEGs at GD18 identified potential ageing-related gene targets for additional study by qPCR. For example, RNAseq DEGs (indicated by *) *Grin2a, Grin2b, Itpr1, and Plcb1* interact with *Gsk3b* and *Psen1* assessed by qPCR (indicated by #) in aged brain. These interact with one another in the “Alzheimer’s disease” (WikiPathways) network. Aged brains of postpartum and non-postpartum mice were analyzed separately, with fold-change revealing change given sertraline administration. A) Interactions in the “Alzheimer’s disease” (WikiPathways) network are shown by type and B) strength of protein–protein interaction (PPI) profile. Modified from the STRING database (string-db.org).

**Supplementary Figure 3:** Volumetric analyses of brain regions revealed no differences by postpartum status nor sertraline consumption in aged animals. Contour tracing of A) aged cortex, B) hippocampus, C) dentate gyrus, D) combined CA regions 1 through 4, E) and corpus callosum. Brain regions defined as described in the Allen Mouse Brain Atlas (<https://mouse.brain-map.org/static/atlas>).

**Supplementary Figure 4:** In postpartum aged animals, distance to target on Day 3 of Barnes Maze was significantly and negatively correlated with A) cortex *Psen1* expression (linear regression, r^2^=0.598, p=0.041) and B) cortex *Csnk2a2* expression (r^2^=0.743, p=0.013), and C) was trending towards significance and negative correlation with cortex *Csnk1a1* expression (r^2^=0.419, *p*=0.116). In non-postpartum aged animals, distance to target on Day 3 of Barnes Maze was not significantly correlated with D) *Psen1* E), *Csnk2a2* F), nor *Csnk1a1* expression.

**Supplementary Figure 5:** In gestational day 18 (GD18) cortex A), there was significantly decreased expression of *Mef2c* (*p*=0.002), *Kalrn* (*p*=0.003), *Ptk2b* (*p*=0.021), *Stx1a* (*p*=0.002); significantly increased expression of *Synpo* (*p*=0.035) and *Atp2b1* (*p*=0.015); and decreased expression of *S100a8* (*p*=0.012), *Camp* (*p*=0.036), and trend-decreased expression of *Grid2ip* (*p*=0.077), all by unpaired two-tail t-test compared to pregnant, water-treated controls. In nonpregnant age-matched cortex A), there was trend-increased expression of *Mef2c* (*p*=0.051) and *Synpo* (*p*=0.081); significantly increased *Stx1a* expression (*p*=0.037); trend-decreased expression of *Homer1* (*p*=0.074) and *S100a8* *(p*=0.091); decreased expression of *Grid2ip* (*p*=0.004), *Irs1* (*p*=0.019); and increased expression of *Kcna1* (*p*=0.048), all by unpaired two-tail t-test compared to nonpregnant, water-treated controls. B) In GD18 hippocampus, there was trend-decreased expression of *Psen1* (*p*=0.067), decreased expression of *Synpo* (*p*=0.017) and *Csnk1a1* (*p*=0.006) by unpaired two-way t-test. No differences were observed in the non-pregnant group. Hashed lines depict control group mean (fold change = 1).

**Supplementary Figure 6:** A principal component analysis (PCA) reveals clustering of RNA sequencing samples after A) PVN sequencing of N=3 samples per condition and B) cortical sequencing of N=3 samples per condition. For both, the X-axis explains the principal component capturing the most variance (PC1) and the y-axis explains the second most (PC2). Percentage of the total variance per direction is shown for each principal component.
