# Supplementary figures and images for: Peripartum sertraline impacts maternal neurobehavioral and neurodegenerative mechanisms in pregnant and postpartum mice
Source: Mol Psychiatry. 2025 Jul 18;30(11):5108–20. doi: 10.1038/s41380-025-03094-x (PMC12532605; doi:10.1038/s41380-025-03094-x)

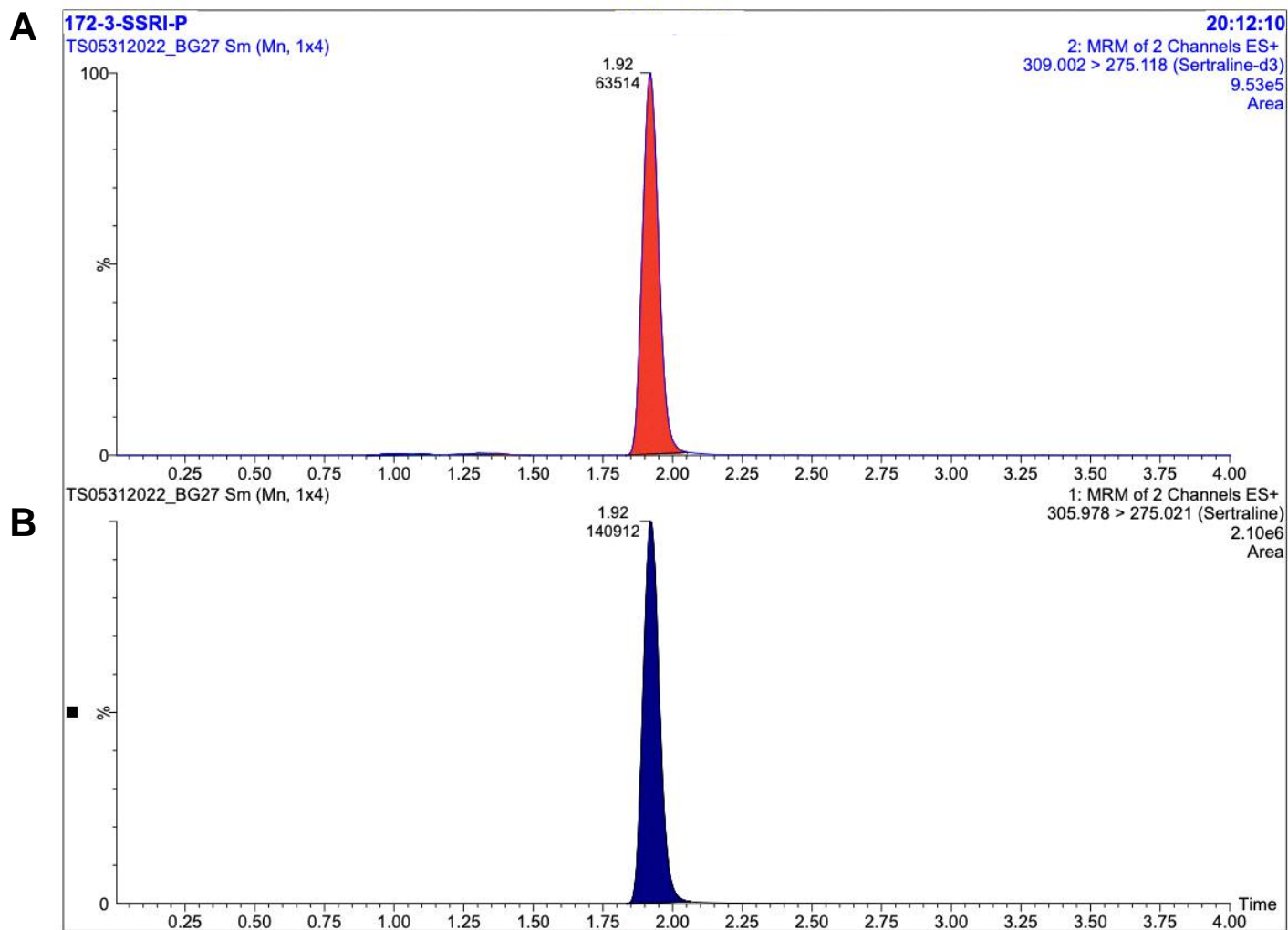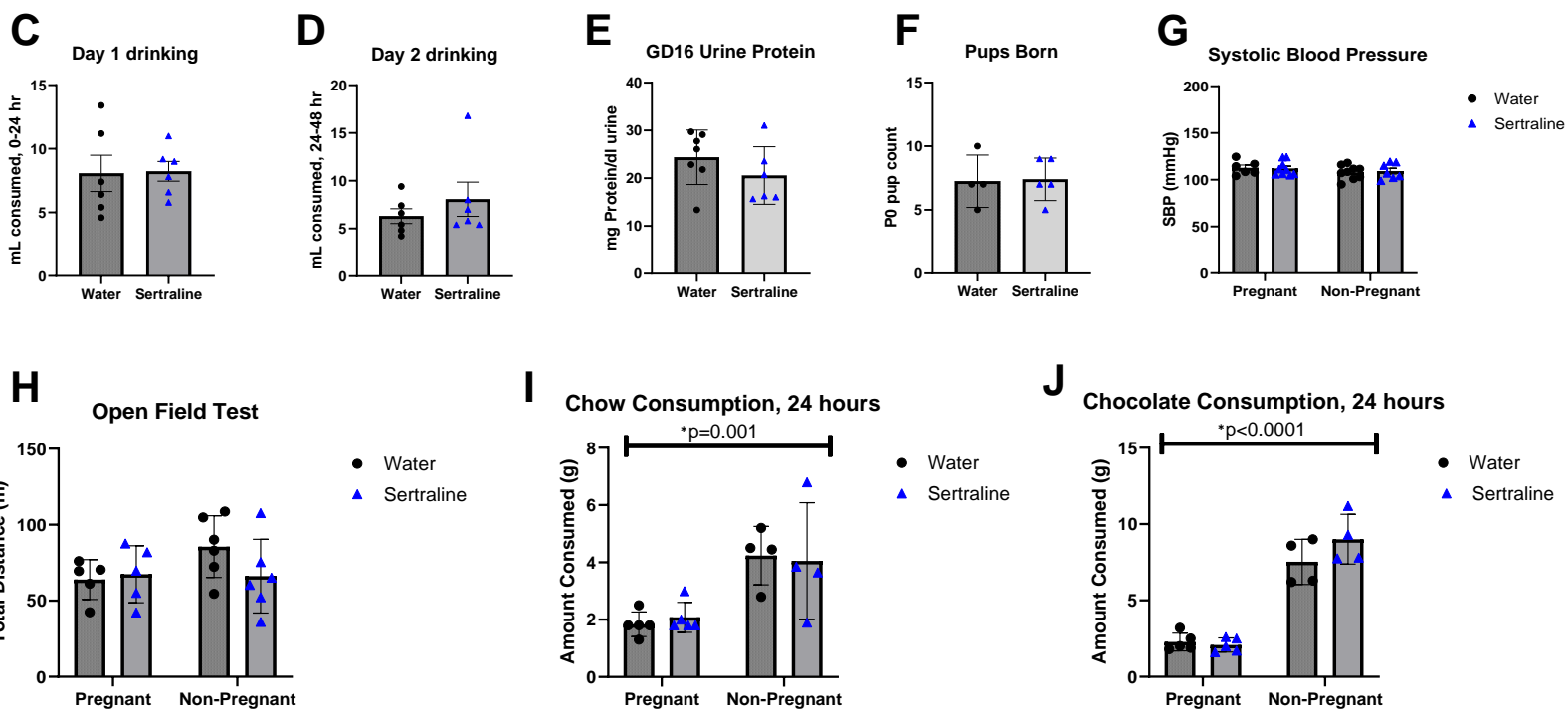

Supplement: Supplementary file 3 — Supplementary figure 1 [file 41380_2025_3094_MOESM3_ESM.pdf]

A

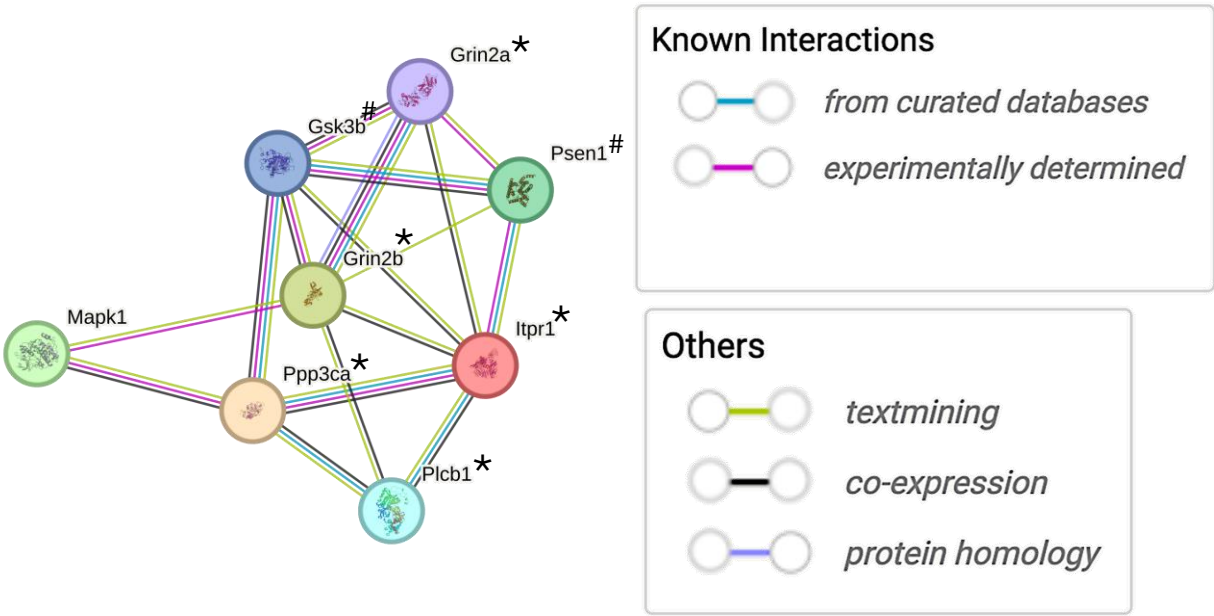

B

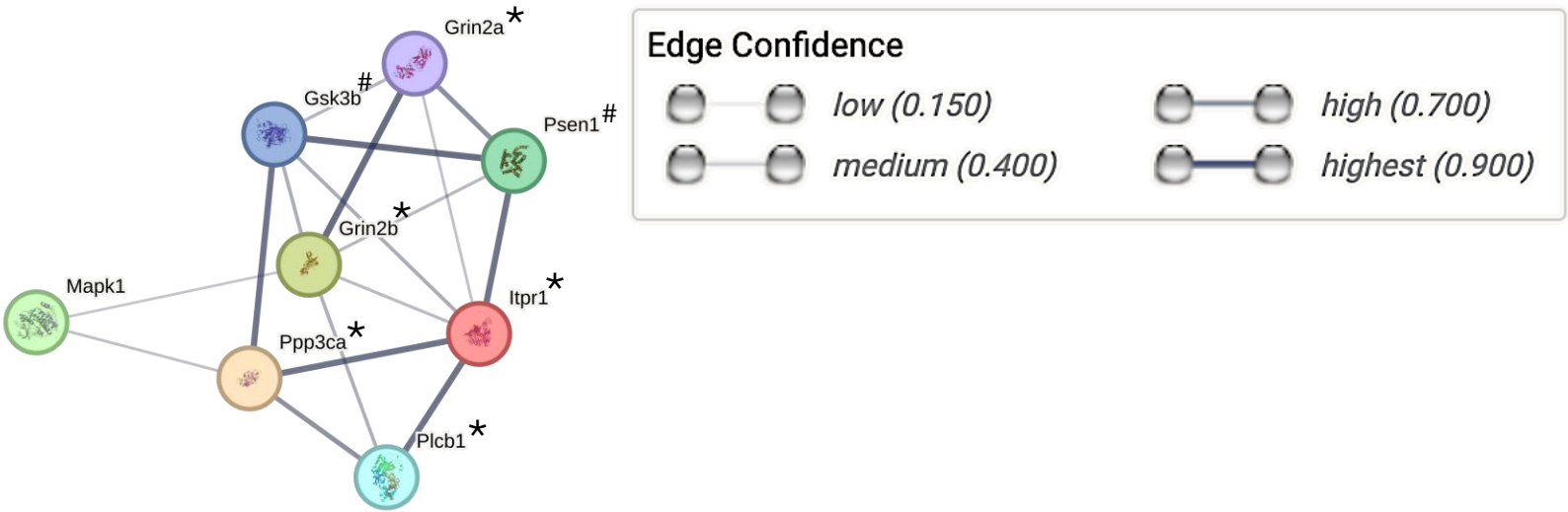

Supplement: Supplementary file 4 — Supplementary figure 2 [file 41380_2025_3094_MOESM4_ESM.pdf]

**A**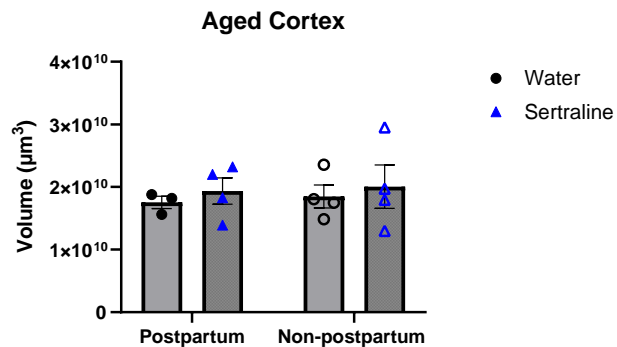**B**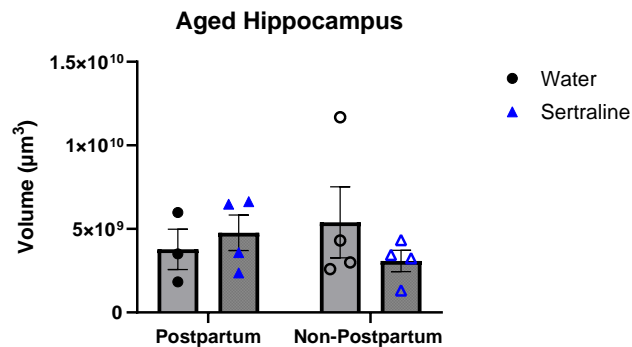**C**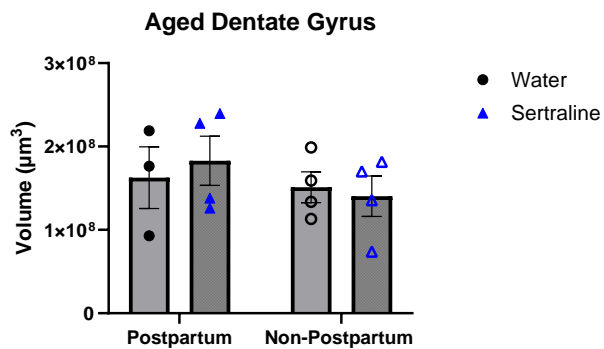**D**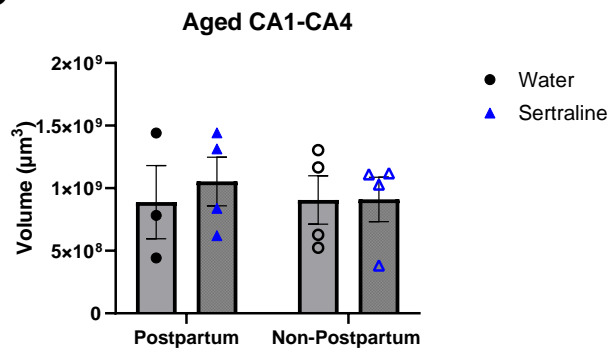**E**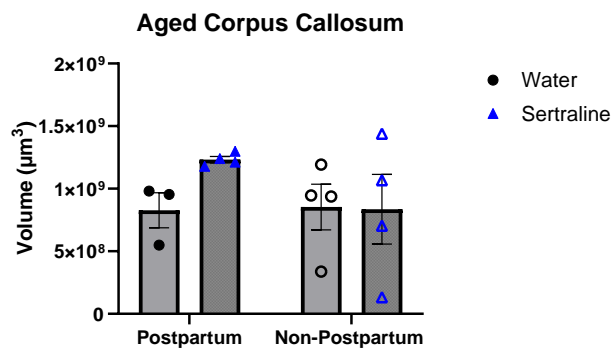

Supplement: Supplementary file 5 — Supplementary figure 3 [file 41380_2025_3094_MOESM5_ESM.pdf]

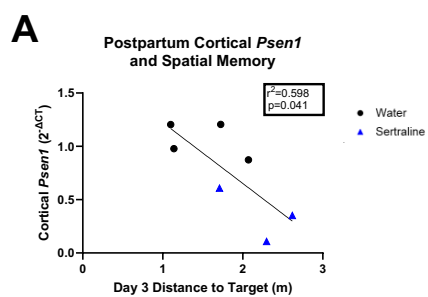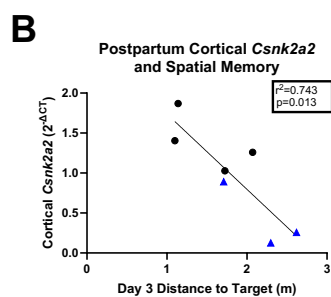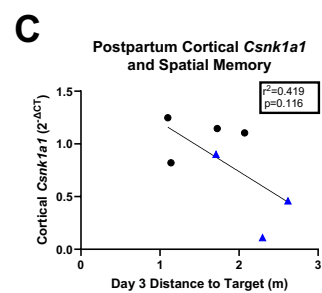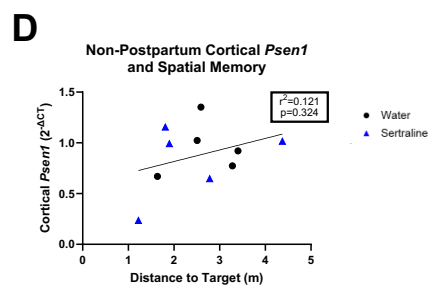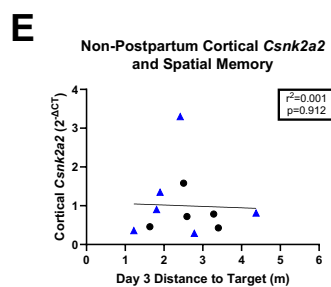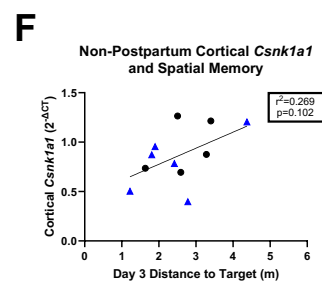

Supplement: Supplementary file 6 — Supplementary figure 4 [file 41380_2025_3094_MOESM6_ESM.pdf]

## A GD18 Cortex: Dementia-Related Targets

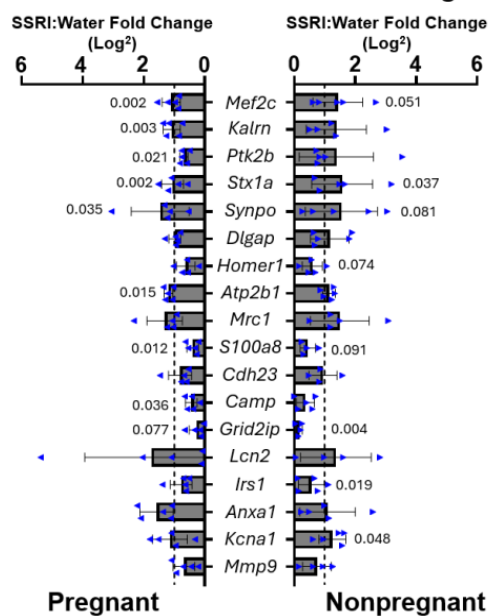

## B GD18 Hippocampus: Dementia-Related Targets

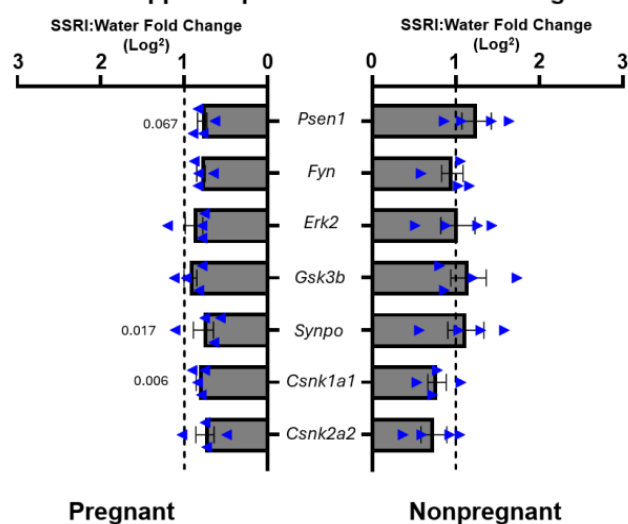

Supplement: Supplementary file 7 — Supplementary figure 5 [file 41380_2025_3094_MOESM7_ESM.pdf]

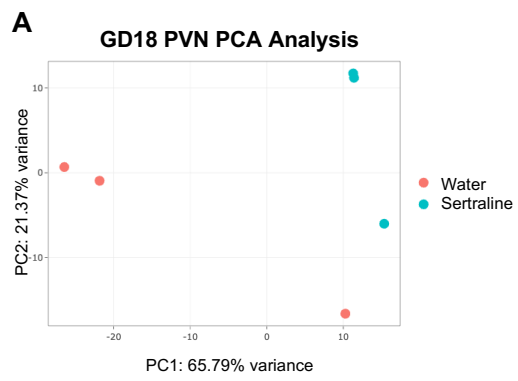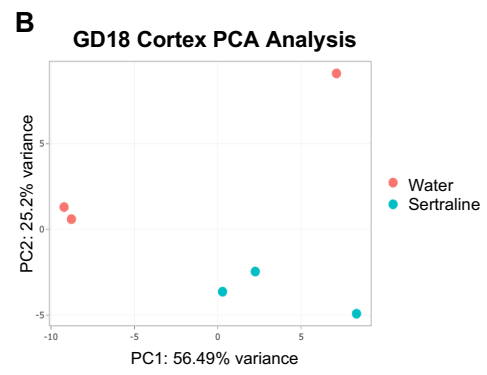

Supplement: Supplementary file 8 — Supplementary figure 6 [file 41380_2025_3094_MOESM8_ESM.pdf]
